# Distribution and Diversity of Comammox *Nitrospira* in Coastal Wetlands of China

**DOI:** 10.3389/fmicb.2020.589268

**Published:** 2020-10-06

**Authors:** Dongyao Sun, Xiufeng Tang, Mengyue Zhao, Zongxiao Zhang, Lijun Hou, Min Liu, Baozhan Wang, Uli Klümper, Ping Han

**Affiliations:** ^1^Key Laboratory of Geographic Information Science (Ministry of Education), School of Geographic Sciences, East China Normal University, Shanghai, China; ^2^State Key Laboratory of Estuarine and Coastal Research, East China Normal University, Shanghai, China; ^3^Institute of Eco-Chongming, East China Normal University, Shanghai, China; ^4^Key Laboratory of Microbiology for Agricultural Environment (Ministry of Agriculture), College of Life Sciences, Nanjing Agricultural University, Nanjing, China; ^5^Institute for Hydrobiology, Technische Universität Dresden, Dresden, Germany

**Keywords:** comammox, *Nitrospira*, estuarine tidal flat wetlands of China, distribution, salinity

## Abstract

Complete ammonia oxidizers (comammox), able to individually oxidize ammonia to nitrate, are considered to play a significant role in the global nitrogen cycle. However, the distribution of comammox *Nitrospira* in estuarine tidal flat wetland and the environmental drivers affecting their abundance and diversity remain unknown. Here, we present a large-scale investigation on the geographical distribution of comammox *Nitrospira* along the estuarine tidal flat wetlands of China, where comammox *Nitrospira* were successfully detected in 9 of the 16 sampling sites. The abundance of comammox *Nitrospira* ranged from 4.15 × 10^5^ to 6.67 × 10^6^ copies/g, 2.21- to 5.44-folds lower than canonical ammonia oxidizers: ammonia-oxidizing bacteria (AOB) and ammonia-oxidizing archaea (AOA). Phylogenetic analysis based on the alpha subunit of the ammonia monooxygenase encoding gene (*amoA*) revealed that comammox Nitrospira Clade A, mainly originating from upstream river inputs, accounts for more than 80% of the detected comammox *Nitrospira*, whereas comammox *Nitrospira* clade B were rarely detected. Comammox *Nitrospira* abundance and dominant comammox *Nitrospira* OTUs varied within the estuarine samples, showing a geographical pattern. Salinity and pH were the most important environmental drivers affecting the distribution of comammox *Nitrospira* in estuarine tidal flat wetlands. The abundance of comammox *Nitrospira* was further negatively correlated with high ammonia and nitrite concentrations. Altogether, this study revealed the existence, abundance and distribution of comammox *Nitrospira* and the driving environmental factors in estuarine ecosystems, thus providing insights into the ecological niches of this recently discovered nitrifying consortium and their contributions to nitrification in global estuarine environments.

## Introduction

Nitrification, a key process of the biogeochemical nitrogen cycle (Konneke et al., 2005), was for over a century considered to exclusively be a two-step microbial process: first, ammonia is oxidized to nitrite by ammonia-oxidizing archaea (AOA) or ammonia-oxidizing bacteria (AOB) (Purkhold et al., 2000; Konneke et al., 2005), with nitrite then being further oxidized to nitrate by nitrite-oxidizing bacteria (NOB) (Winogradsky, 1890). Based on kinetic theory of optimal metabolic processes, the hypothetical existence of complete ammonia-oxidizing (comammox) microorganisms had been proposed (Costa et al., 2006). Their discovery in 2015 (Daims et al., 2015; van Kessel et al., 2015) redefined this key process of the biogeochemical nitrogen cycle.

In contrast to canonical ammonia oxidizers and NOB, comammox *Nitrospira* were confirmed to genes encoding ammonia monooxygenase (AMO) and hydroxylamine dehydrogenase (HAO) genes for initial ammonia oxidation, as well as nitrite oxidoreductase (NXR) genes necessary for nitrite oxidation (Daims et al., 2015; van Kessel et al., 2015; Camejo et al., 2017). Based on phylogenetic analysis using the 16S rRNA gene, all currently known comammox bacteria are members of the genus *Nitrospira*, which is the most abundant and widespread group of NOB (Daims et al., 2015; Pinto et al., 2016). Nevertheless, phylogenetic analysis based on either, the 16S rRNA or *nxr* genes, is not able to distinguish between comammox *Nitrospira* and classical NOB (Pjevac et al., 2017). Consequently, specific primers targeting the *amoA* gene, encoding the alpha subunit of *amo*, were used to screen for the presence of comammox *Nitrospira* in various environments (Pjevac et al., 2017; Abujabhah et al., 2018), owing to the comammox *Nitrospira amoA* genes forming a distinct cluster from canonical ammonia oxidizers. According to similarity analysis of the *amoA* gene comammox *Nitrospira* were subdivided into clade A (including subclade A1 and A2) and clade B (Daims et al., 2015; Xia et al., 2018).

Metagenomic screening of published databases of environmental samples revealed the existence of comammox *Nitrospira* in a wide variety of natural and engineered ecosystems, such as agricultural soils, forest soil, wastewater treatment plants (WWTPs) and drinking water systems (Daims et al., 2015; Pjevac et al., 2017; Wang et al., 2017b, 2019). Intriguingly, there is so far no evidence supporting the existence of comammox *Nitrospira* in marine environments. However, recent evidence indicates the presence of complete ammonia-oxidizing bacteria in estuarine tidal flat wetlands (Yu et al., 2018), the key transition zone of land and marine interaction. Yet, environmental factors affecting the dynamics of comammox *Nitrospira* in these complex habitats remain poorly understood.

In China, with its large population and rapid development of agriculture and economy, considerable amounts of reactive nitrogen (Nr) are released into the environment. Large amounts of Nr are transported into estuarine and coastal ecosystems through river runoff (Cui et al., 2013; Hou et al., 2015a). Consequently, the estuaries and coastal areas of China have become highly Nr-enriched regions, with extreme eutrophication and the formation of algal blooms regularly appearing (Hou et al., 2015b). Located along the long Chinese coastline, there are dozens of estuaries with variable sizes, spanning tropical, subtropical and temperate zones of distinct temperature from north to south (Cao and Wong, 2007). Temperature has been identified as an important factor affecting the distribution and community structure of ammonia oxidizing microorganisms (Urakawa et al., 2008). Further, in estuarine tidal flat wetlands with large amounts of Nr, the present, ammonia-oxidizing microbes responded with high nitrification rates (Zheng et al., 2014). As relatively recently discovered members of the ammonia oxidizing consortia, comammox *Nitrospira* have not yet been identified in marine environments, and there is lack of knowledge about the dynamics of comammox *Nitrospira* in environments affected by their proximity to marine environments, such as estuarine tidal flat wetlands. Estuarine tidal flat wetland ecosystems possess unique environmental characteristics compared to other ecosystems, due to the interaction of land and sea. Estuaries and adjacent areas often experience tidal changes, salinity intrusions, high Nr concentrations and nutrient pulses, which can have a key impact on the community dynamics of ammonia oxidizers (Zheng et al., 2013). We here aimed at revealing comammox *Nitrospira* abundance and environmental factors affecting their dynamics in this complex habitat.

Consequently, the purpose of this study was to (i) examine the diversity and distribution of comammox *Nitrospira* in the estuarine tidal flat wetlands of China; (ii) determine important environmental factors affecting the dynamics of the community structure of comammox *Nitrospira* in estuarine tidal flat wetland systems under marine and land effects; and (iii) elucidate the occurrence of comammox *Nitrospira* and canonical ammonia oxidizers in coastal ecosystems. This is the first study to systematically investigate the environmental drivers of comammox *Nitrospira* abundance and diversity and its interplay with canonical ammonia oxidizers (AOA and AOB) in this natural environment with high salinity content.

## Materials and Methods

### Study Site and Sampling

**FIGURE 1:**
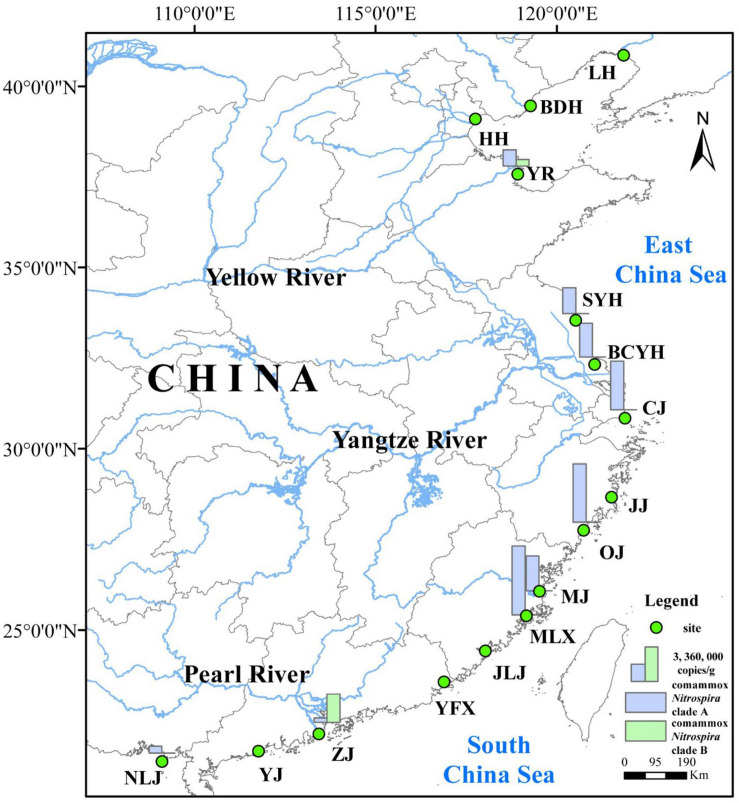
Location of sampling sites in the estuarine tidal flat wetlands of China.

In this study, surface sediment samples were collected from 16 sites along the estuarine tidal flat wetlands of China (Figure 1), including Liaohe (LH), Beidaihe (BDH), Haihe (HH), Yellow River (YR), Sheyanghe (SYH), Bingchayunhe (BCYH), Yangtze River (CJ), and Jiaojiang (JJ), Oujiang (OJ), Minjiang (MJ), Mulanxi (MLX), Jiulongjiang (JLJ), Yifuxi (YFX), Zhujiang (ZJ), Yangjiang (YJ), Nanliujiang (NLJ). Fieldwork was conducted from March to April 2019. At each site, surface sediment samples (0–5 cm) from 6 to 8 plots (50 cm by 50 cm) per site were collected with sterile, stainless steel tubes by S-shaped sampling. The collected sediment samples were stored in sterile plastic bags, sealed, refrigerated and transported back to the laboratory. Upon return to the laboratory, the sediment samples obtained from individual plots at each site were combined at equal proportions inside a sterile plastic bag and mixed using sterile gloves to obtain a homogenized, composite sample for each individual sampling site. Finally, the composite samples were divided into two parts, one was stored at 4°C for the determination of nitrification rates and sediment physicochemical properties, and the second preserved at −20°C for DNA extraction and subsequent molecular analysis.

### Physicochemical Analysis

At each site, *in situ* determination of sediment temperature was carried out with a portable electronic thermometer. A YSI Model 30 salinity meter and a Mettler-Toledo pH meter were used to measure sediment salinity and pH, by mixing sediments with deionized, CO_2_-free water at a sediment to water ratio of 1:2.5 (Lin et al., 2016). Water content of the sediment was measured based on weight loss after drying a known quantity of wet sediment at 80° to constant weight. Sediment particle size was analyzed using a Beckman Coulter LS13320 laser granulometer (United States). Exchangeable ammonium (NH_4_^+^-N), nitrite (NO_2_^–^-N), and nitrate (NO_3_^–^-N) were extracted by adding five parts 2 M KCl to one part of fresh sediment, with the solution subsequently analyzed by spectrophotometry on a continuous-flow nutrient analyzer (SAN Plus, Skalar Analytical B.V., The Netherlands), with detection limits of 0.5 μM for NH_4_^+^-N and 0.1 μM for NO_2_^–^-N and NO_3_^–^-N (Gao et al., 2017). Ferric oxides in sediments were extracted with a mixture of 0.5 M HCl and 0.25 M hydroxylamine hydrochloride and analyzed with the ferrozine-based colorimetric method (Roden and Lovley, 1993; Wei et al., 2020). After treatment with 0.1 M HCl to remove sedimentary carbonate, Organic carbon (OC) was measured on a carbon-hydrogen-nitrogen elementary analyzer (VVarioELIII, Elementary, Germany) (Lin et al., 2017). Total nitrogen (TN) and Total carbon (TC) in sediments were measured using a Carbon nitrogen analyzer (Elementar Vario MAX CN, Germany). All sediment physicochemical parameters were analyzed in triplicate.

### DNA Extraction and PCR Amplification

Total genomic DNA of each sediment sample was extracted in duplicate from 0.5 g of the homogenized sediments using the FastDNA Spin Kit for Soil (QBIOgene, Carlsbad, CA, United States) following the manufacturer’s instruction. Duplicate extracts were combined for down-stream molecular analyses. A NanoDrop-2000 UV-Vis Spectrophotometer (Thermo Scientific) and 1% agarose gel electrophoresis were applied to determine the concentration and quality of extracted DNA. The ammonia monooxygenase alpha subunit encoding genes (*amoA*) of AOA, AOB and comammox *Nitrospira* clade A and comammox *Nitrospira* clade B were amplified by PCR with primers targeting each individual group (Rotthauwe et al., 1997; Pester et al., 2012; Pjevac et al., 2017; Yu et al., 2018). Detailed information about PCR primers used in this study is summarized in Supplementary Table S1. All PCR reactions were performed in a total volume of 20 μL, containing 10 μL 2 × Hieff^®^ PCR Master Mix (Yeasan, China), 1 μL of each primer (10 μM), 1 μL template DNA and 8 μL of ddH_2_O. PCRs were carried out with 5 min (for AOA and AOB) or 8 min (for comammox *Nitrospira*) at 95°C; 35 cycles of 95°C for 30 s, 53°C (for AOA and AOB) or 52°C (for comammox *Nitrospira*) for 30 s, and 72°C for 1 min; and a final 10 min extension cycle at 72°C. During PCR amplifications, negative (without template DNA) and positive controls were always included. DNA extracts obtained from pure cultures of the respective ammonia-oxidizers (*N. nitrosa* 18-3D for AOB, *N. gargensis* for AOA and *N. inopinata* for comammox *Nitrospira*) served as positive controls.

### Real-Time qPCR

To analyze the abundance of AOA, AOB, comammox *Nitrospira* clade A, and comammox *Nitrospira* clade B, quantitative real-time PCR was performed in triplicate with an ABI 7500 sequence detection system (Applied Biosystems, Canada) using the SYBR green quantitative PCR (qPCR) method. Primer pairs, CamoA-19F/616R, amoA-1F/2R, comaAF/R and comaB-244F/659R (Supplementary Table S1), targeting the alpha subunit of the *amo* encoding gene of AOA, AOB, comammox *Nitrospira* clade A and comammox *Nitrospira* clade B, respectively, were used to estimate abundance through qPCR assays. The 21 μL qPCR mixture contained 10 μL of Hieff^®^ qPCR SYBR Green Master Mix with Low Rox Plus (Yeasan, China), 0.4 μL of each primer (10 μM), 1 μL template DNA and 9.2 μL of ddH_2_O. The qPCR was performed with the following protocols: 50°C for 2 min and 95°C for 10 min, followed by 40 cycles of 15 s at 95°C, 30 s at 53°C (for AOA and AOB) and 52°C (for comammox *Nitrospira*), and 40 s at 72°C. To create standard curves, the respective PCR products were purified using the QIAquick PCR Purification Kit (Qiagen, Germany) following the manufacturer’s protocol. The concentration of purified PCR product was estimated with a Nanodrop-2000 Spectrophotometer (Thermo, United States) and copies of purified PCR product calculated as: Copy number = (C × 10^–9^/MW) × NA, with C: template concentration ng/μL, MW: template molecular weight in Daltons, NA: Avogadro’s constant, 6.022 × 10^23^. Then the purified PCR product was serial diluted into a gradient to serve as standards for standard curves. Standard curves with an amplification efficiency 0.94–1.14 and *R*^2^ ≥ 0.99 were accepted and melting curve analysis was performed to assess the amplicon specificity. For each qPCR assay, negative controls containing no template DNA simultaneously analyzed to detect and rule out any potential contamination.

### Potential Nitrification Rates (PNRs)

Potential Nitrification Rates measurements for each sediment sample were carried out in triplicate by referring to the methods previously published by Kurola et al. (2005) and Gao et al. (2016b). In brief, 5 g of sediment and 25 mL of phosphate buffer solution (pH = 7.4: NaCl 8.0 g/L; KCl 0.2 g/L; Na_2_HPO_4_ 0.2 g/L; NaH_2_PO_4_ 0.2 g/L; (NH_4_)_2_SO_4_ 0.132 g/L) were added to 50 mL Falcon tubes. The initial ammonia concentration in this solution was 1 mM. To inhibit nitrite oxidation, 10mM KClO_3_ was added to the solution. Tubes were then incubated in the dark at 25° for 24 h with shaking speed of 120 rpm. After incubation, nitrite was extracted using 2M KCl-solution as described above and analyzed on a continuous flow nutrient analyzer (SAN plus, Skalar Analytical B.V., Netherlands). PNRs were determined through the changes in nitrite concentrations during the incubation period.

### High-Throughput Sequencing and Phylogenetic Analysis

From all samples with positive comammox *Nitrospira*, AOB or AOA detection, PCR products of the *amoA* genes from AOA, AOB and comammox *Nitrospira* were sequenced using Illumina MiSeq by Shanghai Meiji Biomedical Technology Company (Shanghai, China). The raw data was processed using Quantitative Insight into Microbial Ecology (QIIME)^1^ (Caporaso et al., 2010). First, the FLASH plugin was used to stitch paired-end reads based on matched overlapping regions. Using Usearch 7.0^2^, the sequences were clustered to operational taxonomic unit (OTUs) according to 95% nucleic acid similarity and chimeras were eliminated (Schloss, 2013). Sequences of *amoA* gene were analyzed using the BLASTn tool^3^ to select closely related reference sequences. All obtained sequences were aligned using ClustalX (Thompson et al., 1997). Neighbor-joining phylogenetic trees from one representative sequence and its closest reference sequence for each OTU retrieved from GenBank (Kumar et al., 2004) were created using MEGA 7.0 with 1000 bootstrap replicates to evaluate the reliability of the tree topologies (Tamura et al., 2007). The AOA, AOB and comammox sequences obtained in the present study have been deposited in GenBank, with accession numbers MT809785–MT809998, MT797386–MT797560, and MT790359–MT790492.

### Statistical Analysis

.+5The Chao1 species richness and α-diversity indices were calculated in R V3.4 using the Vegan packages V2.5-4 (Oksanen et al., 2013; R Core Team, 2013). The coverage was estimated as the number of observed OTUs divided by the Chao1 species richness estimate (Mohamed et al., 2010). Redundancy analysis (RDA) was performed using the software Canoco 4.5 to evaluate variations in ammonia oxidizing community structure in connection with environmental variables. Correlations between comammox *Nitrospira* clade A OTU abundances and environmental variables were explored with canonical correspondence analysis (CCA). The maximum gradient length determined by detrended correspondence analysis (DCA) in Canoco 4.5 was higher than 4 standard deviations (SD) for ammonia oxidizers, showing that the environmental variables were unimodal (ter Braak and Smilauer, 2002; Zheng et al., 2014). Similarity and clustering of sediment ammonia-oxidizer communities were explored with principal coordinates analysis (PCoA) (Lozupone et al., 2011). Pearson correlation analyses were conducted to test correlations between diversity, abundance and environmental factors. One-way analysis of variance (ANOVA) tests were performed to compare PNRs rates (Gao et al., 2016a). Significance for all tests was accepted at *p* ≤ 0.05. All statistical analyses were performed using SPSS 22.0.

## Results

### Physicochemical Characteristics of the Sediment Samples

Among the sampling sites, located along the estuarine tidal flat wetlands of China, the latitude varied from 21°34′ to 40°51′ N, while longitude varied from 109°05′ to 121°53′ E (Figure 1). The physicochemical characteristics of the sediment samples varied significantly across the different sampling locations (Supplementary Table S2): The sediment temperature was between 12 and 29.9°, salinity ranged from 0.14 to 8.80 ppt and sediment pH varied from 6.41 to 8.91. Sediments were mainly composed of clay, silt, and smaller amounts of sands, with the average particle size ranging from 6.2 to 136.2 μm and water content ranging from 21 to 54%. Sediments consisted of 0.38–2.87% TC, 0.24–3.18% TOC, and 0.04–0.31% TN. Among the observed Nr concentrations, ammonia (6.51–53.76 μg/g) was significantly and positively correlated with nitrate (1.80–11.97 μg/g; *r* = 0.356, *p* < 0.05, *n* = 48) and nitrite (0.14–0.90 μg/g; *r* = 0.404, *p* < 0.01, *n* = 48). Further, concentrations of Fe^2+^ (0.14–0.69 mg/g) were significantly negative correlated with Fe^3+^ (0.16–0.86 mg/g; *r* = 0.336, *p* < 0.05, *n* = 48) (Supplementary Figure S1).

### Potential Nitrification Rates

PNRs in the estuarine tidal flat wetland samples varied from as low as 8.84 up to 174.32 nmol N L^–1^h^–1^ (Supplementary Figure S2), with significant differences in PNRs in estuaries in different latitudes (*p* < 0.05): PNRs of estuaries located in central latitudes were generally higher (97.44 ± 48.67 nmol N L^–1^h^–1^) than those found in northern (42.10 ± 40.79 nmol N L^–1^h^–1^) and southern latitudes (46.49 ± 25.58 nmol N L^–1^h^–1^). PNRs were further positively correlated to pH (*r* = 0.316, *p* < 0.05, *n* = 48) and Fe^2+^ concentrations (*r* = 0.445, *p* < 0.01, *n* = 48). Significant negative correlations were found between the PNRs and salinity (*r* = -0.519, *p* < 0.01, *n* = 48), particle size (*r* = -0.375, *p* < 0.01, *n* = 48) as well as ammonia (*r* = -0.459, *p* < 0.01, *n* = 48). However, between temperature and PNRs no significant relationship (*p* > 0.05) was observed. Unsurprisingly, PNRs were significantly correlated with the abundances of each of the three ammonium oxidizing groups: AOA (*r* = 0.324, *p* < 0.05, *n* = 48), AOB (*r* = 0.582, *p* < 0.01, *n* = 48) and comammox *Nitrospira* (*r* = 0.770, *p* < 0.01, *n* = 48).

### Abundance of Comammox *Nitrospira* and Canonical Ammonia Oxidizers

In the ammonia oxidizing community comammox *Nitrospira* was significantly less abundant than canonical ammonia- oxidizers (Supplementary Figure S3). While AOA and AOB were detected in all tested sediment samples, comammox *Nitrospira* were detected in only 9 of the 16 samples. Among those 9 samples, all contained Comammox *Nitrospira* clade A *amoA*, with abundances between 4.15 × 10^5^ and 6.67 × 10^6^ copies/g dry soil. Comammox *Nitrospira* clade B *amoA* was only detected in 2 samples, but dominated comammox *Nitrospira* abundance in these samples (6.28 × 10^5^–4.01 × 10^6^ copies/g dry soil). Comammox *Nitrospira* was widespread in most parts of the tested wetland areas, and their abundance showed spatial patterns, similar to those detected for the PNRs, with higher abundance in the central (9.41 × 10^6^ ± 1.28 × 10^6^ copies/g dry soil) than southern (2.77 × 10^6^ ± 2.53 × 10^6^ copies/g dry soil) and northern (1.55 × 10^6^ ± 6.3 × 10^5^ copies/g dry soil) latitudes (Figure 4). The highest copy number of comammox *Nitrospira amoA* genes was detected at central latitude site MLX (6.66 × 10^6^ copies/g dry soil), and the lowest one was recorded at the most southern site NLJ (6.47 × 10^5^ copies/g dry soil). Again, no significant correlation with temperature (*p* > 0.05), but a significant positive correlation with Fe_2_C (*r* = 0.403, *p* < 0.01, *n* = 27) and a negative correlation with salinity (*r* = -0.321, *p* < 0.05, *n* = 27) were detected (Supplementary Figure S1), further indicating the strong effect of salinity and metal ions on ammonia oxidation.

Among the canonical ammonia oxidizers, which were detected in all samples, abundance ranged from 1.15 × 10^6^ to 3.66 × 10^7^ copies/g dry soil (AOA) and 1.76 × 10^5^ to 1.73 × 10^7^ copies/g dry soil (AOB) (Supplementary Figure S3). In 10 of the 16 estuarine tidal flat wetland samples AOA showed higher abundance than AOB (Supplementary Figure S4). The abundance of AOA was positively correlated with temperature (*r* = 0.44, *p* < 0.01, *n* = 48) with highest abundance in estuaries of central and southern latitudes. Contrary, AOB were mainly distributed across the central and northern latitudes, and dominated ammonia oxidizer abundances at the northern latitudes.

### Diversity of Comammox *Nitrospira*

In total 69,858 high-quality comammox *Nitrospira amoA* gene sequences were generated from the 9 samples where comammox *Nitrospira* were detected. Comammox *Nitrospira amoA* gene sequences were clustered into 118 OTUs based on 95% nucleotide similarity, with the number of OTUs for each sample ranges from 17 to 70. Chao1 richness estimates ranged from 17.5 (for site BCYH) to 76.9 (for site CJ), with coverage for comammox *Nitrospira* detection ranging from 89–100%. Salinity was the only environmental factor showing significant negative effects (*r* = -0.768, *p* < 0.05, *n* = 9) on the Chao1 richness as well as the Shannon diversity of comammox *Nitrospira* communities (*r* = 0.698, *p* < 0.05, *n* = 9). In addition, TC was positively correlated with Shannon diversity (*r* = 0.793, *p* < 0.05, *n* = 9) while not affecting richness (Supplementary Table S4).

**FIGURE 2:**
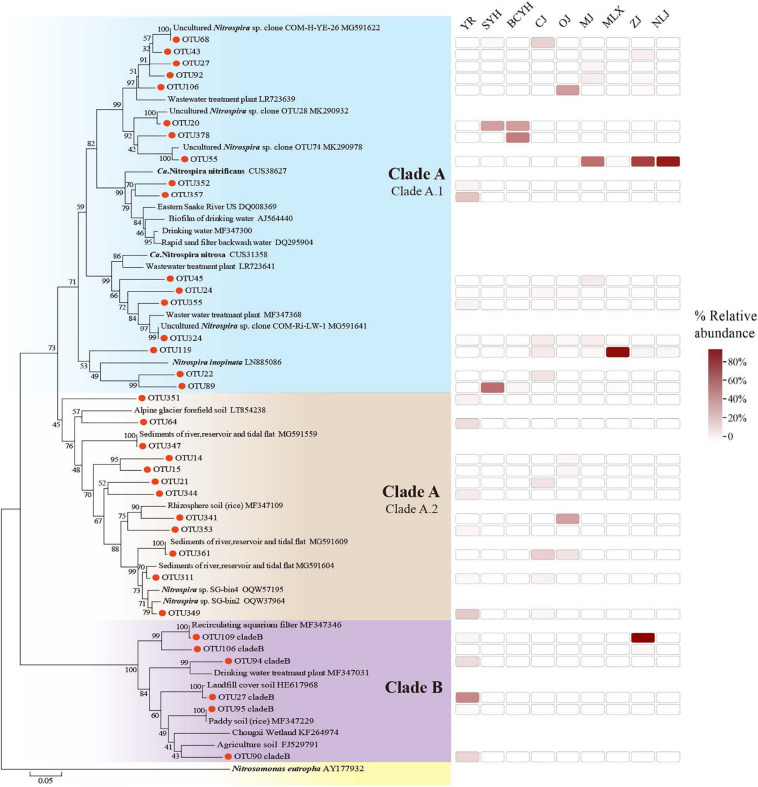
Phylogenetic tree of comammox *Nitrospira amoA* genes constructed by neighbor joining methods. Sequences were collected from this study and the GenBank database. Bootstrap values are given on the branches of the neighbor-joining tree. The scale bar represents 5% nucleic acid sequence divergence.

The phylogenetic tree (Figure 2) generated for the comammox *Nitrospira amoA* gene supported the division of comammox *Nitrospira* into two clades (clade A: 118, clade B: 16). Clade A can further be subdivided into clade A1 and A2 (Xia et al., 2018). Clade A1 had slightly more representative OTUs (68/118 clade A OTUs) than clade A2 (50/118), however among the 50 most abundantly detected comammox *Nitrospira* OTUs, 43 (86%) belonged to clade A1. Clade B was present in 2 samples only (YR and ZJ). Again, a latitudinal pattern emerged on the OTU abundance level: While OTU55 and OTU119 dominated comammox *Nitrospira* abundance in the southern latitude estuaries, a wider variety of OTUs were observed in those estuaries from central or northern latitude (Supplementary Figure S5).

Based on the same methodology for canonical ammonia-oxidizers, 214 AOA and 175 AOB, OTUs were detected. Most AOA sequences (107 OTUs) were divided into the *Nitrosopumilus* cluster (group1.1b), while 94 OTUs belonged to the *Nitrososphaera* cluster (group1.1a) (Supplementary Figure S6). The remaining OTUs were affiliated with the *Nitrosotalea* cluster (group1.1a – associated). Although the number of OTUs in group 1.1a was slightly lower than in group 1.1b, 78.53% of the detected sequences were affiliated with group 1.1a. For AOB, all sequences were grouped within known species of *Betaproteobacteria*, *Nitrosomonas* (119 OTUs) and *Nitrosospira* (56 OTUs). More than half of the AOB sequences belonged to the 6 *Nitrosomonas*-related clusters, and grouped with the lineages *N. communis, N. europaea*, *N. oligotropha*, *N*. *marina*, *N*. *cryotolerans*, and *N*. sp. Nm143. The *Nitrosospira* were subdivided into 4 clusters and a single *Nitrosospira-like* strain (Supplementary Figure S7). Base on Pearson correlation analysis, none of the environmental factors showed significant effects on the Shannon diversity within both AOA and AOB communities (Data not shown).

### The Effects of Environmental Factors

**FIGURE 3:**
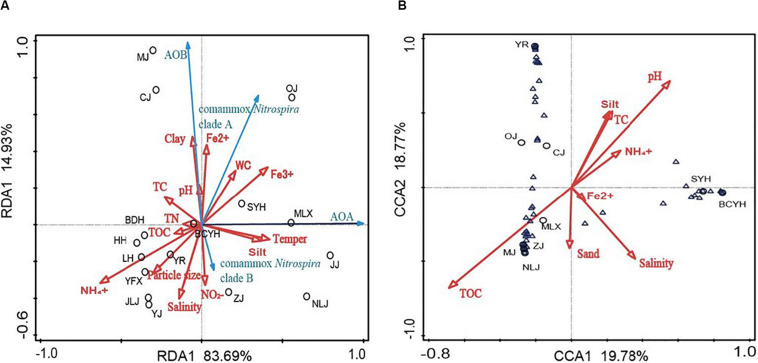
**(A)** RDA plot based on the relative abundances of all ammonia-oxidizers (qPCR results); **(B)** CCA plot based on comammox *Nitrospira* clade A OTU abundances (high-throughput sequencing results). Black circles: samples, Blue arrows: ammonia-oxidizers, Blue triangles: Comammox *Nitrospira* clade A OTUs, Red arrows: environmental factors

Redundancy analysis was performed to evaluate variations in ammonia oxidizing community structure in connection with environmental variables (Figure 3A). The first two RDA dimensions explained 98.62% of the cumulative variance. Higher temperatures were promoting the growth of AOA, but had no significant effect on AOB and comammox *Nitrospira* abundance. Different forms of iron ions significantly promoted the abundance of ammonia-oxidizers, and ammonia, particle size and pH were also showing varying degrees of influence on their abundance (Figure 3A).

**FIGURE 4:**
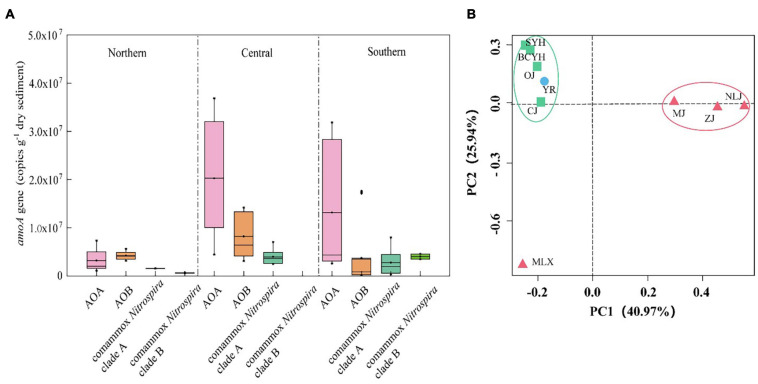
**(A)** Abundance of ammonia-oxidizers in the distinct areas based on qPCR results. **(B)** UniFrac weighted PCoA analysis of comammox *Nitrospira* communities in the estuary tidal flat wetlands of China. Red triangle: Southern estuaries (MJ, ZJ, MLX, NLJ); Green square: Central estuaries (BCYH, SYH, CJ, OJ); Blue circle: Northern estuaries (YR).

The correlations of comammox *Nitrospira* clade A OTU abundances in the different communities with environmental variables were tested by CCA. The first two CCA dimensions accounted for 38.55% of the cumulative variance of the comammox *Nitrospira* community environmental correlation (Figure 3B). Comammox *Nitrospira* clade A community diversity in the sediments of Chinese estuarine tidal flat wetlands were significantly correlated to TOC (*P* = 0.004, *F* = 1.6), pH (*P* = 0.04, *F* = 1.5) and salinity (*P* = 0.013, *F* = 1.5), accounted for half of the total expositive power. Despite the contribution of other environmental variables (including ammonia levels, TN, particle size) were not significant (*P* > 0.05), they also contributed considerably to the CCA’s expositive power.

## Discussion

### Distribution of Comammox *Nitrospira* in Estuarine Tidal Flat Wetlands of China

**FIGURE 5:**
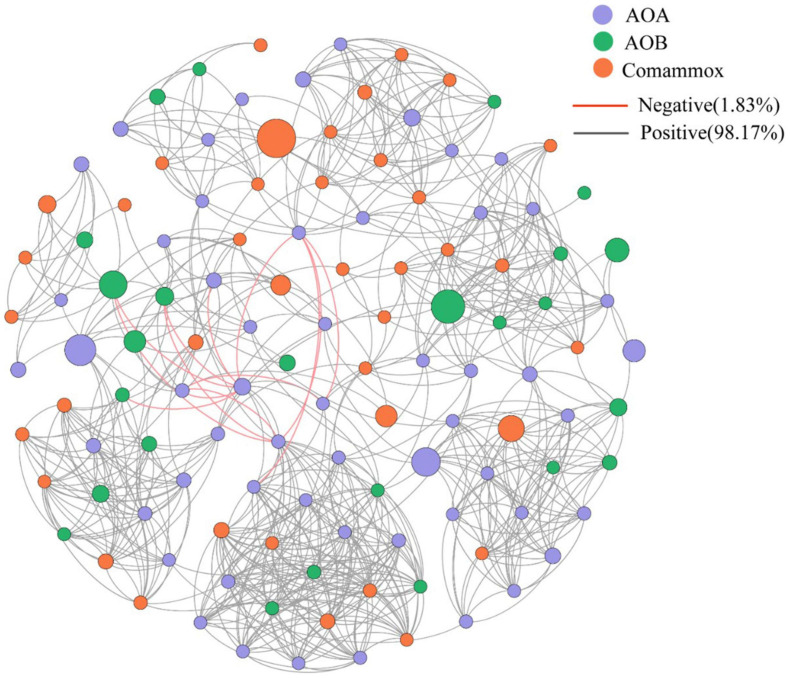
Network analysis of all ammonia oxidizers. Different colored circles represent different ammonia oxidants, orange lines represent negative interaction, black lines represent positive interaction.

Comammox *Nitrospira* were detected from 9 of the 16 sampling sites. The abundance of comammox *Nitrospira* ranged from 4.15 × 10^5^ to 6.66 × 10^6^ copies/g, 2.21- to 5.44-folds lower than canonical ammonia oxidizers: AOA and AOB, which were both detected at every sampling location. The three types of microorganisms use ammonia as an energy substance, and hence are in direct nutrient competition. However, they are able to coexist in most environments. In the estuarine tidal flat wetlands nitrifying microbial network (AOA, AOB, and comammox *Nitrospira*) (Figure 5), the correlation between all species is mainly positive (98.17%) and their abundance is equally correlated with the detected PNRs. The average ratio of comammox *Nitrospira* to AOA and AOB is 0.18 and 0.46. From the proportion of abundance, the contribution of comammox *Nitrospira* to the PNRs and hence nitrification may be smaller than that of AOA and AOB. The abundance of AOA was higher than that of AOB in 10 of the 16 sediment samples, with the AOA/AOB ratio ranging from 0.22 up to 205. No significant decreases of PNRs could be observed in intertidal sediment after AOB were inhibited by ampicillin, implying that AOA might play the most important role for the nitrification potential in this specific ecosystem (Zheng et al., 2014).

Recent studies of ammonia oxidizing microbial communities based on *amoA* genes revealed that relative proportions of comammox *Nitrospira*, AOB and AOA are highly variable across environmental matrixes including both natural and engineered systems (Table 1). While in most ecosystems (e.g., river water, grassland, agricultural and paddy soil) AOA or AOB were the dominating organisms (Kits et al., 2017; Zhang et al., 2019), comammox *Nitrospira* were found to mainly dominate in engineered systems (Bartelme et al., 2017; Tatari et al., 2017), but also in some natural environments, such as specific freshwater lakes (Shi et al., 2020) or the river water and sediments of the Yangtze River (Liu et al., 2020). Contrary, no marine comammox organisms have been identified so far.

**TABLE 1:** Distribution of ammonia-oxidizers in different ecosystems.

Country	Ecosystem	AOA	AOB	comammox *Nitrospira* clade A	comammox *Nitrospira* clade B	References
America	Recirculating aquaculture systems	0.94 × 10^8^–3.4 × 10^8^ (copies/g)	2.6 × 10^3^–5.0 × 10^5^ (copies/g)	1.6 × 10^8^–4.2 × 10^8^ (copies/g)	–	Bartelme et al., 2017
Denmark	Drinking water	1.2 × 10^3^–3.4 × 10^3^ (copies/m^3^)	1.6 × 10^7^–10.0 × 10^7^ (copies/m^3^)	0.82 × 10^8^–2.58 × 10^8^ (copies/m^3^)	–	Tatari et al., 2017
Austria	Waste water treatment plant	–	1.3 × 10^3^–2.1 × 10^3^ (copies/ng DNA)	3.4 × 10^2^–6.8 × 10^2^ (copies/ng DNA)	–	Pjevac et al., 2017
China	Overlying water in river	3.34 × 10^3^–2.18 × 10^7^ (copies/L)	1.06 × 10^5^–2.98 × 10^7^ (copies/L)	1.25 × 10^4^ (copies/L)	–	Zhang et al., 2019
China	Agriculture soil	–	–	4.14 × 10^4^–1.65 × 10^7^ (copies/g)	9.44 × 10^2^–2.12 × 10^6^ (copies/g)	Xu et al., 2020
Italy	Rice paddy soil	2.1 × 10^3^–3.1 × 10^3^ (copies/ng DNA)	–	3.6 × 10^2^–4.6 × 10^2^ (copies/ng DNA)	3.5 × 10^2^–4.5 × 10^2^ (copies/ng DNA)	Pjevac et al., 2017
Italy	Forest soil	1.4 × 10^2^–2.6 × 10^2^ (copies/ng DNA)	1.7 × 10^3^–3.5 × 10^3^ (copies/ng DNA)	–	2.9 × 10^2^–4.9 × 10^2^ (copies/ng DNA)	Pjevac et al., 2017
China	River sediment	1.84 × 10^2^–3 × 10^2^ (copies/ng DNA)	9.3 × 10^1^–3.4 × 10^3^ (copies/ng DNA)	1.8 × 10^2^–2.8 × 10^2^ (copies/ng DNA)	–	Zhao et al., 2019
China	Intertidal sediment	1.7 × 10^2^–4.9 × 10^3^ (copies/ng DNA)	2.2 × 10^2^–5.4 × 10^3^ (copies/ng DNA)	1.6 × 10^2^–3.2 × 10^2^ (copies/ng DNA)	–	Zhao et al., 2019
**China**	**Estuary tidal wetland sediment**	**1.15 × 10^6^–1.66 × 10^7^ (copies/g) or 5.71 × 10^1^–6.27 × 10^3^ (copies/ng DNA)**	**1.76 × 10^5^–1.73 × 10^7^ (copies/g) or 1.05 × 10^1^–1.57 × 10^3^ (copies/ng DNA)**	**4.15 × 10^5^–6.67 × 10^6^ (copies/g) or 2.74 × 10^1^–7.02 × 10^2^ (copies/ng DNA)**	**6.28 × 10^5^–4.01 × 10^6^ (copies/g) or 1.1 × 10^2^–2.65 × 10^2^ (copies/ng DNA)**	**This study**

Here, comammox *Nitrospira amoA* sequences obtained from the estuarine tidal flat wetland of China were affiliated with those previously identified in terrestrial sources from China (Yu et al., 2018; Zhao et al., 2019). Comammox *Nitrospira* clade A1 mainly found in fresh water, and engineered ecosystems (Pjevac et al., 2017; Zhao et al., 2019) was more abundant and widely distributed than clade A2 that mainly occupies agriculture soils (Xu et al., 2020). The lower abundance of comammox *Nitrospira* clade B compared comammox *Nitrospira* clade A observed in this study was also in line with previous studies (Pjevac et al., 2017; Xu et al., 2020). Overall, this indicates that the main source of comammox *Nitrospira* in the estuarine tidal flat wetland ecosystem is upstream river runoff input with only minor contributions from soil and sediments (clade A2 and clade B).

### The Influence of Environmental Factors on the Abundance and Structure of Comammox *Nitrospira* Flora

The major environmental factors associated with comammox *Nitrospira* abundance and defining comammox *Nitrospira community* structure were identified as pH, salinity, the availability of iron ions, TOC, ammonia, nitrite and particle size.

The pH in the study area ranged between 6.74 and 8.65, and displayed a positive correlation with comammox *Nitrospira* abundance. Blum et al. (2018) found ammonia oxidizers to preferentially grow in slightly alkaline environments, due to the optimal pH range (7–8) for key nitrifying enzymes (e.g., *amo* and *hao*). Although the *amo* enzyme of comammox *Nitrospira is* different from that of traditional AOB (Palomo et al., 2016), it still showed similar pH adaptability. While some studies have shown that pH will impact the niche distinction between different ammonia-oxidizing groups, the number of comammox *Nitrospira* found in low pH environments such as forest soils (pH < 6.0) can on occasion still exceed that of AOA and AOB (Hu and He, 2017). No significant effect of pH on AOA and AOB has been shown in this study, so the exact effect of environmental pH on *amo* of comammox *Nitrospira* in comparison with canonical ammonia oxidizer enzymes remains to be determined.

Salinity displayed a significant negative correlation with comammox *Nitrospira* abundance and PNRs, as well as comammox *Nitrospira* community diversity. Oceans were previously speculated to not provide a suitable habitat for any known comammox *Nitrospira*, based on the higher salinity levels (Kuypers, 2017; Santos et al., 2017). Here, the highest richness of comammox *Nitrospira is* detected in the estuaries with lowest salinity in the central latitudes. The highest number of OTU were found in these central estuaries, with 29 of the Top 30 OTUs present and 14 of them dominating. Clade A1 was mostly abundant, but clade A2 was considerably distributed in the lower salinity CJ and OJ estuaries, indicating that clade A1 can adapt to environments with a wider range of salinity, while clade A2 is exclusively adapted to habitats with low salinity.

In the southern estuaries with intermediate salinity, due to strong evaporation rates at high temperature, but also high amounts of river runoff, still considerable comammox *Nitrospira* abundance can be detected. However, the comammox *Nitrospira* community was dominated by exclusively two species. Specifically, OTU 55, which was close to an uncultured *Nitrospira* sp. clone OTU74 discovered by Zhao et al. (2019), was most abundantly found in the southern estuary (MJ, ZJ, NLJ). It dominated the southern comammox *Nitrospira* community together with *Nitrospira* species OTU119, which might be better adapt to the higher-salinity sampling sites in the MLX, clustering with *Nitrospira inopinata* (Daims et al., 2015).

Moreover, comammox *Nitrospira* was not detected in areas with higher salinity (e.g., HH 8.80 ppt and LH 5.92 ppt). These northern rivers have small amounts of river runoffs and are mostly affected by the ocean, which might be the reason that no comammox *Nitrospira* was detected in samples from these areas. An exception in this area was the Yellow River, which is an aboveground river with an estuary located higher than sea level, hence less affected by the ocean. Intriguingly, the Yellow River sediment was the only one hosting comammox *Nitrospira* clade A2 and clade B, mainly associated with soil and sediment ecosystems (Yu et al., 2018). This correlates with the large amount of sediment input in the upper Yellow River and might further mask the influence of the higher salinity generally observed in the northern regions. Although there were differences in the dominant OTU species in the central and northern estuaries, the overall community structure was not significantly different, while there are differences with the community structure of the southern estuaries, which was also verified by PCoA analysis (Figure 4B).

Iron exists in the active center of various enzymes in nitrifying bacteria, which is involved in the transport of molecular oxygen and the transformation of nitrogen (Wang et al., 2013). It was found in this study that Fe^2+^ has a positive correlation with the PNRs, and abundance of comammox *Nitrospira*. Fe^2+^ acts on enzymes (e.g., *amo, nxr*) in the metabolic nitrification process and as part of various cofactors and proteins that are beneficial to nitrification. Although there are differences in *amo* between AOB and comammox *Nitrospira*, Fe^2+^ can increase the microbial activity and increase the nitrification reaction by increasing the enzyme activity of *amo* (Ren et al., 2011) for both AOB and comammox *Nitrospira.* Interestingly, Fe^3+^ showed a positive correlation with the abundance of AOA, indicating different impact mechanisms on archaea. The cells of most nitrifying organisms have complex inner membrane pleated structures (flaky, vesicular and tubular), and iron as a chemical catalyst can increase the permeability of the cell membrane, thereby accelerating the transmission rate of nutrients (Wang et al., 2003), which could be a potential mode of effect.

It has been shown that in low oxygen environments, high TOC concentrations will mutually strengthen the effect on nitrification (Zhang et al., 2002). While oxygen concentrations in the estuarine tidal flats were indeed low, no effect of TOC on the PNRs was found in this study. However, the community structure of comammox *Nitrospira* was affected by TOC to a certain extent. The complex interaction mechanism between comammox *Nitrospira* and TOC requires further investigation.

Both AOA and comammox *Nitrospira* displayed significant negative correlation with ammonia in this study, indicating better adaptions to oligotrophic environments. Levels of ammonia are known to affect various nitrifying microorganisms during the nitrification process (Santoro, 2016). The affinity for ammonia is much higher in AOA than AOB (Martens-Habbena et al., 2009), and comammox *Nitrospira* species *Nitrospira inopinata* has been shown to possess a higher affinity for ammonia than non-marine AOA and many AOB, resulting in a competitive advantage at low ammonia concentrations (Kits et al., 2017). Due to the large accumulation of reactive nitrogen in estuarine tidal flat wetlands the higher concentrations of ammonia might have put comammox *Nitrospira* at a competitive disadvantage as nitrogen affinity did not play a significant role.

A negative correlation between comammox *Nitrospira* and the availability of nitrite was found, suggesting that low levels of nitrite may be beneficial for comammox *Nitrospira*. Comammox *Nitrospira* is phylogenetically closely related to lineage II NOB, but, other than for NOB, nitrite is not a necessary substrate or final metabolite of comammox *Nitrospira*. Comammox *Nitrospira* have, compared with canonical NOB, a poor affinity for nitrite (Kits et al., 2017) and might thus have a competitive disadvantage against these strictly nitrite oxidizing bacteria in this environment rich in reactive nitrogen.

The final observed factor, particle size, controls the physicochemical characteristics of the sediments which can subsequently affect the structure of the microbiome (Dang et al., 2010), but the specific influence mechanism of particle size on comammox *Nitrospira* is not obvious.

### Distribution of Canonical Ammonia Oxidizers

Among the canonical ammonia oxidizers AOB generally had a lower diversity of *amoA* genes than AOA in the estuarine tidal flat wetlands (Supplementary Table S4), was consistent with studies on other estuarine and coastal environments (Jin et al., 2011; Zheng et al., 2014). Temperature was positively correlated with AOA abundance. Significant correlations were also found between salinity and the distribution, but not the abundance, of AOA and AOB. AOB is more abundant than AOA in the northern estuaries with higher salinity, whereas AOA dominated in the regions with lower salinity and higher temperatures, previous studies also verified this result (Mosier and Francis, 2008; Bernhard et al., 2010).

Ammonia-oxidizing archaea detected in the study were divided into two major development branches, sediment group1.1a and soil group1.1b. 78.5% of the AOA sequence obtained belongs to sediment group1.1a, which is the dominant group. Clustered with AOA from sedimentary environments such as estuaries and oceans (Bernhard et al., 2010; Zheng et al., 2013), it is widely distributed in all regions. The remaining 21.5% of the sequences belong to group 1.1b, clustered with AOA in soil environments such as paddy soil and irrigated desert soil (Wang et al., 2015, 2017a), mainly distributed in the north and south estuaries of higher salinity.

Ammonia-oxidizing bacteria is divided into 6 *Nitrosomonas* branches and 2 *Nitrosospira* branches. *Nitrosomonas* under branch OTU637 was the most dominant AOB group in the sediments, accounting for 19.9% of the total sequences. It showed high homology with *Nitrosomonas* sp. Nm143 isolated from saltmarsh sediment (Purkhold et al., 2003; Bernhard et al., 2019), explaining its wide distribution of higher salinity estuaries in the north and south. Among *Nitrosospira* species, we found that a *Nitrosospira*-like branch occupies a dominant position with 89% similarity to the other four pure culture clusters, clustered with some sediment samples in the estuary environment (Jin et al., 2011); further analysis of this strain branch is required. From cluster 3 most OTUs were found exclusively in the Yellow River estuary in the northern region, which implies the particularity of AOB distribution in this estuary, potentially based on the introduction of soil in the upper Yellow river.

## Conclusion

Comammox *Nitrospira* was found widely distributed in the estuarine tidal flat wetland of China, but with lower abundances and diversity compared to canonical AOB and AOA. Comammox *Nitrospira* communities were dominated by clade A1, indicating input of river runoff as the main source. The abundance and diversity of comammox *Nitrospira* in tidal flat wetlands of estuaries of China were larger in the central parts than in the South or the North, which was consistent with observed PNRs. The comammox *Nitrospira* community structures were different in southern, central and northern China estuaries. In general, comammox *Nitrospira* is distributed more in estuaries with high runoff and lower salinity. For estuarine tidal flat wetland systems, salinity, pH and iron were important factors affecting the distribution of comammox *Nitrospira*. Ammonia, nitrite and TOC also influence the distribution of comammox *Nitrospira* to some extent. Our findings provide new insights into the distribution of comammox *Nitrospira* in the natural ecosystem, was an important supplement to the biogeochemical Nitrogen cycle.

## Data Availability Statement

The datasets presented in this study can be found in online repositories. The names of the repository/repositories and accession number(s) can be found in the article/Supplementary Material.

## Author Contributions

PH and DS contributed to the development of the research plan and project goals. DS, XT, and MZ performed laboratory work, and contributed to data analysis. XT and ZZ participated in the analysis of the sequencing data. LH, ML, and PH contributed to sources of project funding. BW and UK helped with the data interpretation. DS and PH wrote the manuscript with the input of all other authors. All authors contributed to the article and approved the submitted version.

## Conflict of Interest

The authors declare that the research was conducted in the absence of any commercial or financial relationships that could be construed as a potential conflict of interest.

## Footnotes

1
http://qiime.org/

2
http://drive5.com/uparse/

3
http://www.ncbi.nlm.nih.gov/BLAST
