# Corrigendum: Distribution and Diversity of Comammox *Nitrospira* in Coastal Wetlands of China

**DOI:** 10.3389/fmicb.2021.731921

**Published:** 2021-08-26

**Authors:** Dongyao Sun, Xiufeng Tang, Mengyue Zhao, Zongxiao Zhang, Lijun Hou, Min Liu, Baozhan Wang, Uli Klümper, Ping Han

**Affiliations:** ^1^Key Laboratory of Geographic Information Science (Ministry of Education), School of Geographic Sciences, East China Normal University, Shanghai, China; ^2^State Key Laboratory of Estuarine and Coastal Research, East China Normal University, Shanghai, China; ^3^Institute of Eco-Chongming, East China Normal University, Shanghai, China; ^4^Key Laboratory of Microbiology for Agricultural Environment (Ministry of Agriculture), College of Life Sciences, Nanjing Agricultural University, Nanjing, China; ^5^Institute for Hydrobiology, Technische Universität Dresden, Dresden, Germany

**Keywords:** comammox, *Nitrospira*, estuarine tidal flat wetlands of China, distribution, salinity

In the original article, there was a mistake in Figure 1, *Location of sampling sites in the estuarine tidal flat wetlands of China*. as published. There was a mistake in the scale label. The corrected Figure 1 appears below.

**Figure 1:**
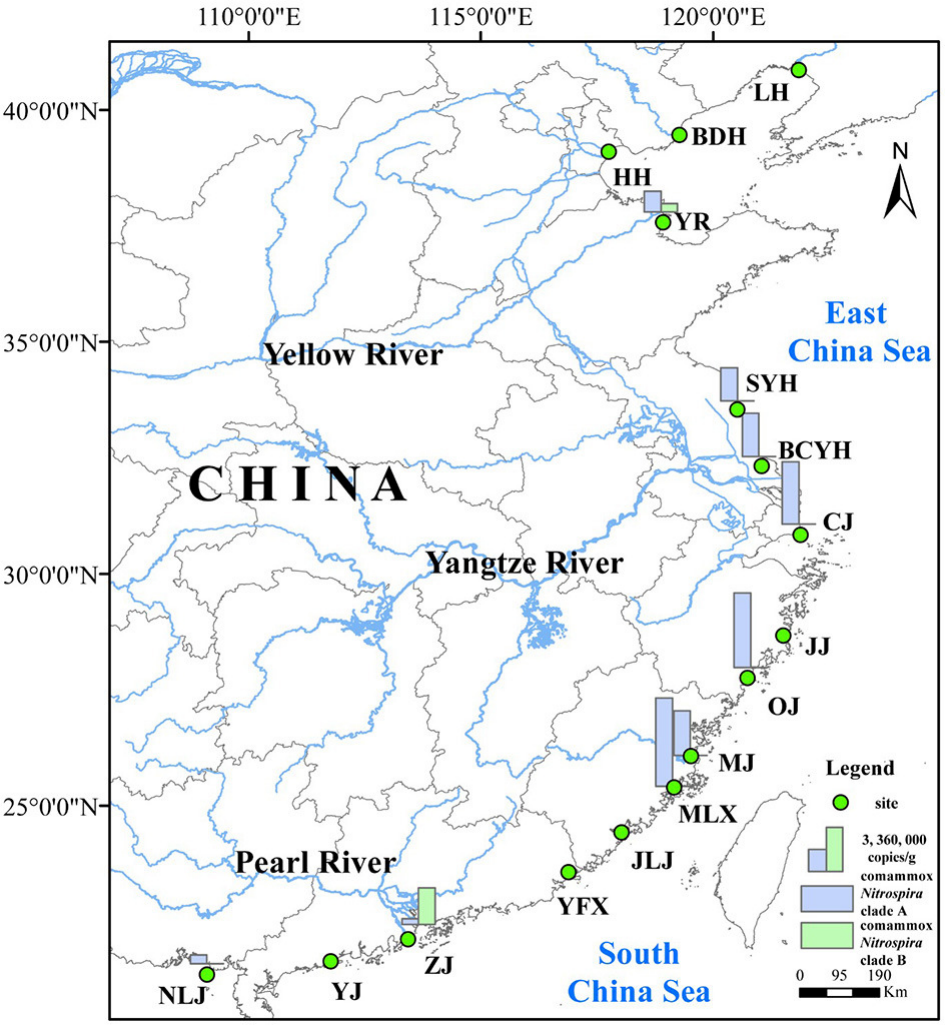
Location of sampling sites in the estuarine tidal flat wetlands of China.

In the original article, there was a mistake in Figure 4, *(A) Abundance of ammonia-oxidizers in the distinct areas based on qPCR results*. as published. There was a mistake in the values on the Y-axis. The corrected Figure 4 appears below.

**Figure 4:**
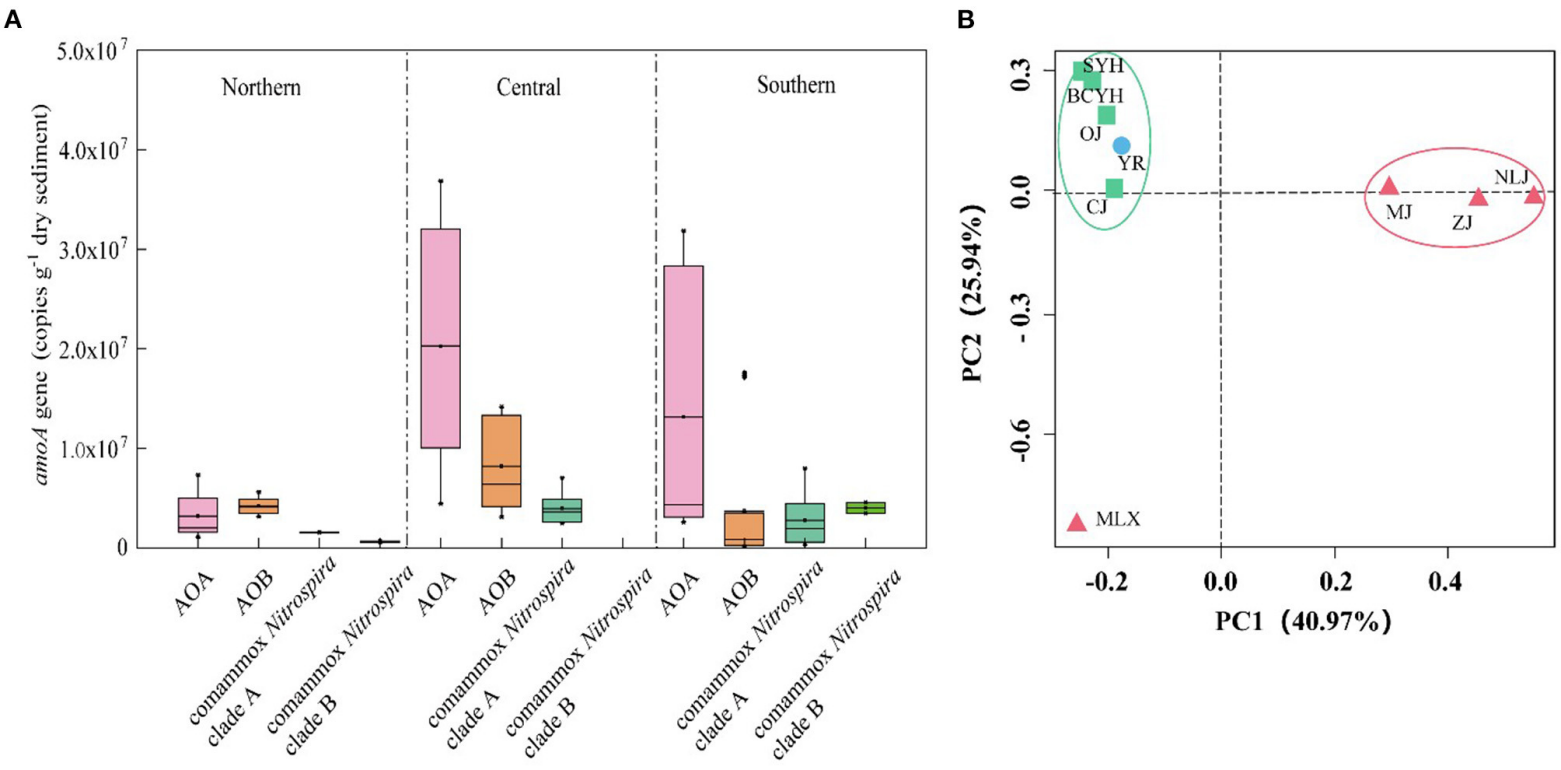
**(A)** Abundance of ammonia-oxidizers in the distinct areas based on qPCR results. **(B)** UniFrac weighted PCoA analysis of comammox Nitrospira communities in the estuary tidal flat wetlands of China. Red triangle: Southern estuaries (MJ, ZJ, MLX, NLJ); Green square: Central estuaries (BCYH, SYH, CJ, OJ); Blue circle: Northern estuaries (YR).

In the original article, there was a mistake in Figure 5, *Network analysis of all ammonia oxidizers. Different colored circles represent different ammonia oxidants, orange lines represent negative interaction, black lines represent positive interaction*. as published. The colors indicating negative and positive interactions were not clearly shown. The corrected Figure 5 appears below.

**Figure 5:**
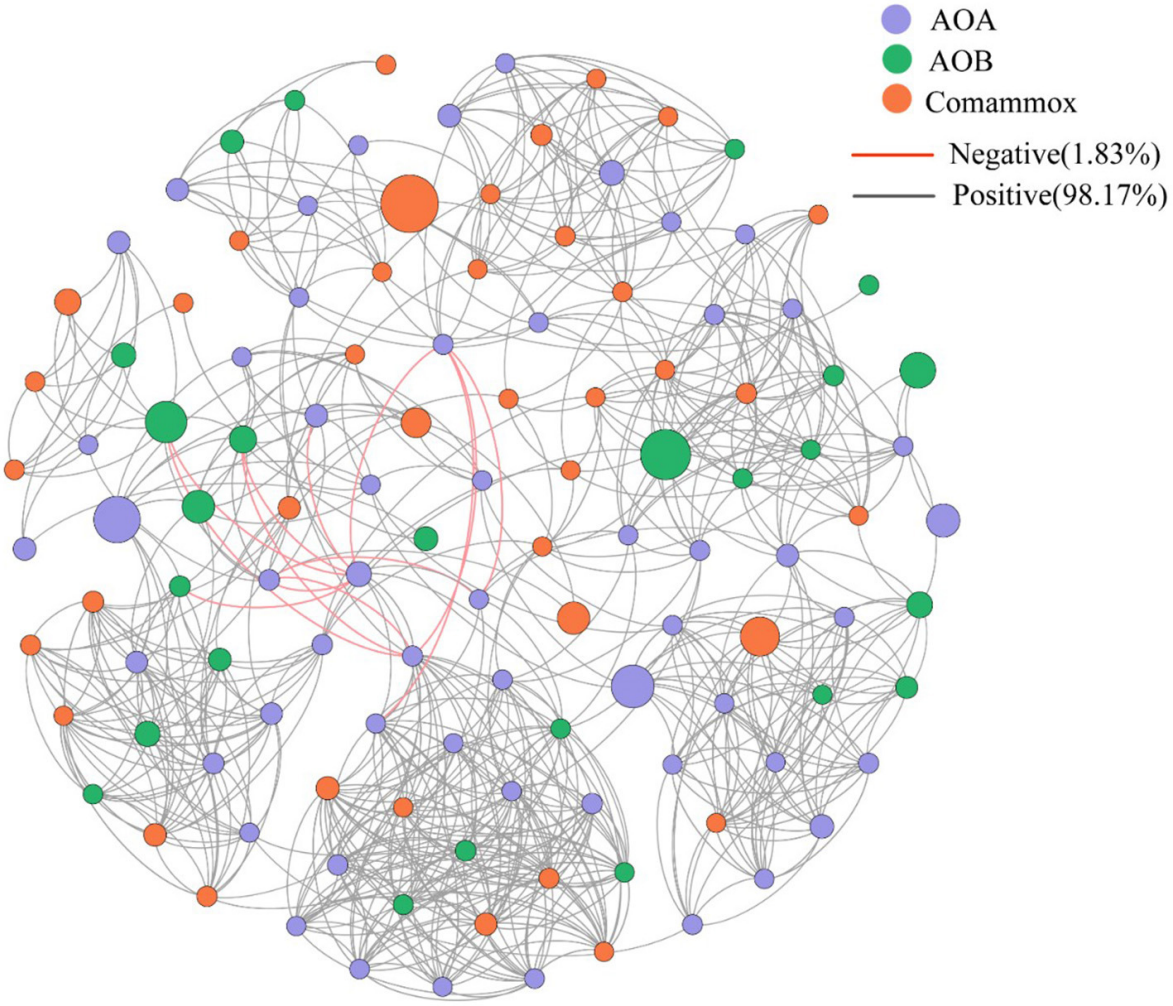
Network analysis of all ammonia oxidizers. Different colored circles represent different ammonia oxidants, orange lines represent negative interaction, black lines represent positive interaction.

In the original article, there was a mistake in Table 1, *Distribution of ammonia-oxidizers in different ecosystems*. as published. The values and units were not correctly indicated in the Table. The corrected Table 1 appears below.

**Table 1:** Distribution of ammonia-oxidizers in different ecosystems.

**Country**	**Ecosystem**	**AOA**	**AOB**	**comammox *Nitrospira* clade A**	**comammox *Nitrospira* clade B**	**References**
America	Recirculating aquaculture systems	0.94 × 10^8^-3.4 × 10^8^ (copies/g)	2.6 × 10^3^-5.0 × 10^5^ (copies/g)	1.6 × 10^8^-4.2 × 10^8^ (copies/g)	–	Bartelme et al., 2017
Denmark	Drinking water	1.2 × 10^3−^3.4 × 10^3^ (copies/m^3^)	1.6 × 10^7^-10.0 × 10^7^ (copies/m^3^)	0.82 × 10^8^-2.58 × 10^8^ (copies/m^3^)	–	Tatari et al., 2017
Austria	Waste water treatment plant	–	1.3 × 10^3^-2.1 × 10^3^ (copies/ng DNA)	3.4 × 10^2^-6.8 × 10^2^ (copies/ng DNA)	–	Pjevac et al., 2017
China	Overlying water in river	3.34 × 10^3^-2.18 × 10^7^ (copies/L)	1.06 × 10^5^-2.98 × 10^7^ (copies/L)	1.25 × 10^4^ (copies/L)	–	Zhang et al., 2019
China	Agriculture soil	–	–	4.14 × 10^4^-1.65 × 10^7^ (copies/g)	9.44 × 10^2^-2.12 × 10^6^ (copies/g)	Xu et al., 2020
Italy	Rice paddy soil	2.1 × 10^3^-3.1 × 10^3^ (copies/ng DNA)	–	3.6 × 10^2^-4.6 × 10^2^ (copies/ng DNA)	3.5 × 10^2^-4.5 × 10^2^ (copies/ng DNA)	Pjevac et al., 2017
Italy	Forest soil	1.4 × 10^2^-2.6 × 10^2^ (copies/ng DNA)	1.7 × 10^3^-3.5 × 10^3^ (copies/ng DNA)	–	2.9 × 10^2^-4.9 × 10^2^ (copies/ng DNA)	Pjevac et al., 2017
China	River sediment	1.84 × 10^2^-3 × 10^2^ (copies/ng DNA)	9.3 × 10^1^-3.4 × 10^3^ (copies/ng DNA)	1.8 × 10^2^-2.8 × 10^2^ (copies/ng DNA)	–	Zhao et al., 2019
China	Intertidal sediment	1.7 × 10^2^-4.9 × 10^3^ (copies/ng DNA)	2.2 × 10^2^-5.4 × 10^3^ (copies/ng DNA)	1.6 × 10^2^-3.2 × 10^2^ (copies/ng DNA)	–	Zhao et al., 2019
**China**	**Estuary tidal wetland sediment**	**1.15** **× 10** ^**6**^ **-1.66** **× 10** ^**7**^ **(copies/g) or 5.71** **× 10** ^**1**^ **-6.27** **× 10** ^**3**^ **(copies/ng DNA)**	**1.76** **× 10** ^**5**^ **-1.73** **× ** **10** ^**7**^ **(copies/g) or 1.05** **× 10** ^**1**^ **-1.57** **× ** **10** ^**3**^ **(copies/ng DNA)**	**4.15** **× 10** ^**5**^ **-6.67** **× 10** ^**6**^ **(copies/g) or 2.74** **× 10** ^**1**^ **-7.02** **× 10** ^**2**^ **(copies/ng DNA)**	**6.28** **× 10** ^**5**^ **-4.01** **× 10** ^**6**^ **(copies/g) or 1.1** **× 10** ^**2**^ **-2.65** **× 10** ^**2**^ **(copies/ng DNA)**	**This study**

In the original article, there was an error. There were some errors in the values of copy numbers.

A correction has been made to the Abstract:

Complete ammonia oxidizers (comammox), able to individually oxidize ammonia to nitrate, are considered to play a significant role in the global nitrogen cycle. However, the distribution of comammox *Nitrospira* in estuarine tidal flat wetland and the environmental drivers affecting their abundance and diversity remain unknown. Here, we present a large-scale investigation on the geographical distribution of comammox *Nitrospira* along the estuarine tidal flat wetlands of China, where comammox *Nitrospira* were successfully detected in 9 of the 16 sampling sites. The abundance of comammox *Nitrospira* ranged from 4.15 × 10^5^ to 6.67 × 10^6^ copies/g, 2.21- to 5.44-folds lower than canonical ammonia oxidizers: ammonia-oxidizing bacteria (AOB) and ammonia-oxidizing archaea (AOA). Phylogenetic analysis based on the alpha subunit of the ammonia monooxygenase encoding gene (*amoA*) revealed that comammox Nitrospira Clade A, mainly originating from upstream river inputs, accounts for more than 80% of the detected comammox *Nitrospira*, whereas comammox *Nitrospira* clade B were rarely detected. Comammox *Nitrospira* abundance and dominant comammox *Nitrospira* OTUs varied within the estuarine samples, showing a geographical pattern. Salinity and pH were the most important environmental drivers affecting the distribution of comammox *Nitrospira* in estuarine tidal flat wetlands. The abundance of comammox *Nitrospira* was further negatively correlated with high ammonia and nitrite concentrations. Altogether, this study revealed the existence, abundance and distribution of comammox *Nitrospira* and the driving environmental factors in estuarine ecosystems, thus providing insights into the ecological niches of this recently discovered nitrifying consortium and their contributions to nitrification in global estuarine environments.

In the original article, there was an error. There were some errors in the values of copy numbers.

A correction has been made to ***RESULTS, Abundance of Comammox Nitrospira and Canonical Ammonia Oxidizers***, *Paragraph 1*:

In the ammonia oxidizing community comammox *Nitrospira* was significantly less abundant than canonical ammonia-oxidizers (Supplementary Figure 3). While AOA and AOB were detected in all tested sediment samples, comammox *Nitrospira* were detected in only 9 of the 16 samples. Among those 9 samples, all contained Comammox *Nitrospira* clade A *amoA*, with abundances between 4.15 × 10^5^ and 6.67 × 10^6^ copies/g dry soil. Comammox *Nitrospira* clade B *amoA* was only detected in 2 samples, but dominated comammox *Nitrospira* abundance in these samples (6.28 × 10^5^-4.01 × 10^6^ copies/g dry soil). Comammox *Nitrospira* was widespread in most parts of the tested wetland areas, and their abundance showed spatial patterns, similar to those detected for the PNRs, with higher abundance in the central (9.41 × 10^6^ ±1.28 × 10^6^ copies/g dry soil) than southern (2.77 × 10^6^ ± 2.53 × 10^6^ copies/g dry soil) and northern (1.55 × 10^6^ ± 6.3 × 10^5^ copies/g dry soil) latitudes (Figure 4). The highest copy number of comammox *Nitrospira amoA* genes was detected at central latitude site MLX (6.66 × 10^6^ copies/g dry soil), and the lowest one was recorded at the most southern site NLJ (6.47 × 10^5^ copies/g dry soil). Again, no significant correlation with temperature (*p* > 0.05), but a significant positive correlation with Fe_2_C (*r* = 0.403, *p* < 0.01, *n* = 27) and a negative correlation with salinity (*r* = −0.321, *p* < 0.05, *n* = 27) were detected (Supplementary Figure 1), further indicating the strong effect of salinity and metal ions on ammonia oxidation.

In the original article, there was an error. There were some errors in the values of copy numbers.

A correction has been made to ***RESULTS, Abundance of Comammox Nitrospira and Canonical Ammonia Oxidizers***, *Paragraph 2*:

Among the canonical ammonia oxidizers, which were detected in all samples, abundance ranged from 1.15 × 10^6^ to 3.66 × 10^7^ copies/g dry soil (AOA) and 1.76 × 10^5^ to 1.73 × 10^7^ copies/g dry soil (AOB) (Supplementary Figure 3). In 10 of the 16 estuarine tidal flat wetland samples AOA showed higher abundance than AOB (Supplementary Figure 4). The abundance of AOA was positively correlated with temperature (*r* = 0.44, *p* < 0.01, *n* = 48) with highest abundance in estuaries of central and southern latitudes. Contrary, AOB were mainly distributed across the central and northern latitudes, and dominated ammonia oxidizer abundances at the northern latitudes.

In the original article, there was an error. There were some errors in the values of copy numbers.

A correction has been made to ***DISCUSSION, Distribution of Comammox Nitrospira in Estuarine Tidal Flat Wetlands of China***, *Paragraph 1*:

Comammox *Nitrospira* were detected from 9 of the 16 sampling sites. The abundance of comammox *Nitrospira* ranged from 4.15 × 10^5^ to 6.66 × 10^6^ copies/g, 2.21- to 5.44-folds lower than canonical ammonia oxidizers: AOA and AOB, which were both detected at every sampling location. The three types of microorganisms use ammonia as an energy substance, and hence are in direct nutrient competition. However, they are able to coexist in most environments. In the estuarine tidal flat wetlands nitrifying microbial network (AOA, AOB, and comammox *Nitrospira*) (Figure 5), the correlation between all species is mainly positive (98.17%) and their abundance is equally correlated with the detected PNRs. The average ratio of comammox *Nitrospira* to AOA and AOB is 0.18 and 0.46. From the proportion of abundance, the contribution of comammox *Nitrospira* to the PNRs and hence nitrification may be smaller than that of AOA and AOB. The abundance of AOA was higher than that of AOB in 10 of the 16 sediment samples, with the AOA/AOB ratio ranging from 0.22 up to 205. No significant decreases of PNRs could be observed in intertidal sediment after AOB were inhibited by ampicillin, implying that AOA might play the most important role for the nitrification potential in this specific ecosystem (Zheng et al., 2014).

## Publisher's Note

All claims expressed in this article are solely those of the authors and do not necessarily represent those of their affiliated organizations, or those of the publisher, the editors and the reviewers. Any product that may be evaluated in this article, or claim that may be made by its manufacturer, is not guaranteed or endorsed by the publisher.
