# Supplementary material for: Distribution and Diversity of Comammox Nitrospira in Coastal Wetlands of China
Source: Front Microbiol. 2020 Oct 6;11:589268. doi: 10.3389/fmicb.2020.589268 (PMC7573150; doi:10.3389/fmicb.2020.589268)
Supplement: Supplementary file 1 [file Data_Sheet_1.docx]

Supplementary Material

**Table. S1** Primers used for PCR and qPCR.

| **Target gene** | **Primer name** | **Primer sequence (5’-3’)** | **Length (bp)** | **Reference** |
| --- | --- | --- | --- | --- |
| comammox *Nitrospira* clade A *amoA* | comaAF | TGCGGIGACTGGGAYTTC | 514 | Yu et al. (2018) |
|  | comaAR | AGATCATAGTGCTRTGICC |  |  |
| comammox *Nitrospira* clade B *amoA* | comaB-244F | TAYTTCTGGACGTTCTA | 415 | Pjevac et al. (2017) |
|  | comaB-659R | TAYTTCTGGACATTCTA |  |  |
| AOA *amoA* | CamoA-19F | ATGGTCTGGCTWAGACG | 629 | Perster et al. (2012) |
|  | CamoA-616R | GCCATCCATCTGTATGTCCA |  |  |
| AOB *amoA* | amoA-1F | GGGGTTTCTACTGGTGGT | 491 | Rotthauwe et al. (1997) |
|  | amoA-2R | CCCCTCKGSAAAGCCTTCTTC |  |  |

**Table S2** Environmental parameters for the estuarine tidal flat wetlands of China samples analyzed in this study

| **Sample ID** | **Salinity**  **(ppt)** | **Tempe rature**  **(℃)** | **pH** | **Particle size**  **(**μ**m)** | **Clay**  **(%)** | **Silt**  **(%)** | **Sand**  **(%)** | **Water content**  **(%)** | **NH_4_^+^**  **(**μ**g/g)** | **NO_2_^-^**  **(**μ**g/g)** | **NO_3_^-^**  **(**μ**g/g)** | **Fe^2^**  **(mg/g)** | **Fe^3^**  **(mg/g)** | **TN**  **(%)** | **TC**  **(%)** | **TOC**  **(%)** |
| --- | --- | --- | --- | --- | --- | --- | --- | --- | --- | --- | --- | --- | --- | --- | --- | --- |
| **LH** | 8.80 | 11.50 | 8.25 | 10.42 | 4.71 | 92.98 | 2.31 | 40.46 | 53.76 | 0.61 | 9.71 | 0.62 | 0.21 | 0.11 | 1.26 | 0.95 |
| **BDH** | 3.66 | 14.85 | 7.41 | 72.28 | 3.69 | 43.27 | 53.03 | 40.48 | 33.99 | 0.18 | 11.97 | 0.25 | 0.33 | 0.12 | 1.03 | 1.16 |
| **HH** | 5.92 | 19.05 | 8.38 | 11.35 | 8.26 | 86.32 | 5.43 | 26.63 | 42.63 | 0.53 | 8.43 | 0.44 | 0.19 | 0.11 | 1.70 | 1.03 |
| **YR** | 0.14 | 15.55 | 8.56 | 13.71 | 2.63 | 93.71 | 3.66 | 32.21 | 30.10 | 0.44 | 9.78 | 0.22 | 0.31 | 0.06 | 1.94 | 0.30 |
| **SYH** | 2.28 | 16.20 | 8.65 | 17.82 | 3.75 | 79.90 | 16.36 | 27.10 | 11.12 | 0.20 | 7.69 | 0.37 | 0.34 | 0.04 | 1.47 | 0.24 |
| **BCYH** | 1.64 | 21.80 | 8.43 | 12.76 | 4.92 | 91.99 | 3.09 | 32.63 | 32.77 | 0.36 | 7.26 | 0.62 | 0.35 | 0.08 | 1.67 | 0.43 |
| **CJ** | 0.66 | 26.20 | 8.08 | 10.70 | 5.88 | 90.54 | 3.58 | 39.09 | 14.42 | 0.24 | 8.85 | 0.69 | 0.41 | 0.14 | 1.90 | 0.98 |
| **JJ** | 1.35 | 22.20 | 8.91 | 9.92 | 6.63 | 93.26 | 0.11 | 41.75 | 12.14 | 0.22 | 2.03 | 0.42 | 0.61 | 0.10 | 1.25 | 0.60 |
| **OJ** | 0.33 | 18.85 | 8.25 | 7.72 | 7.10 | 92.88 | 0.02 | 45.18 | 6.94 | 0.15 | 3.07 | 0.67 | 0.45 | 0.12 | 1.01 | 0.84 |
| **MJ** | 0.40 | 16.55 | 6.96 | 10.49 | 41.54 | 23.29 | 35.18 | 47.52 | 17.54 | 0.22 | 3.68 | 0.47 | 0.86 | 0.13 | 1.52 | 1.32 |
| **MLX** | 3.31 | 22.30 | 8.12 | 9.47 | 5.32 | 84.79 | 9.90 | 42.91 | 6.51 | 0.14 | 1.80 | 0.34 | 0.69 | 0.11 | 1.01 | 0.99 |
| **JLJ** | 3.41 | 19.60 | 7.94 | 12.48 | 3.93 | 70.60 | 25.47 | 26.44 | 25.22 | 0.23 | 2.88 | 0.53 | 0.16 | 0.07 | 0.76 | 0.69 |
| **YFX** | 0.40 | 19.85 | 6.41 | 7.88 | 10.57 | 84.47 | 4.96 | 52.37 | 18.62 | 0.29 | 7.63 | 0.14 | 0.74 | 0.31 | 2.87 | 3.18 |
| **ZJ** | 0.87 | 19.75 | 6.74 | 6.19 | 18.92 | 81.01 | 0.07 | 53.69 | 26.90 | 0.23 | 4.78 | 0.60 | 0.62 | 0.19 | 1.39 | 1.43 |
| **YJ** | 2.27 | 23.25 | 7.18 | 136.19 | 1.28 | 20.98 | 77.74 | 20.98 | 27.78 | 0.18 | 2.20 | 0.30 | 0.25 | 0.06 | 0.38 | 0.44 |
| **NLJ** | 2.15 | 30.05 | 6.86 | 9.92 | 10.81 | 78.45 | 10.74 | 34.96 | 11.03 | 0.90 | 11.83 | 0.29 | 0.60 | 0.12 | 1.26 | 1.35 |

Abbreviations: NH_4_^+^, ammonia; NO_3_^-^, nitrate; NO_2_^-^, nitrite; TN, total nitrogen; TC, total carbon; TOC, total organic carbon.

**Table S3** The α-diversity index of all ammonia-oxidizers

|  | comammox | | | | | AOA | | | | | AOB | | | | |
| --- | --- | --- | --- | --- | --- | --- | --- | --- | --- | --- | --- | --- | --- | --- | --- |
| Sample | Chao1 | Shannon | 1/Simpson | Richness | Coverage | Chao1 | Shannon | 1/Simpson | Richness | Coverage | Chao1 | Shannon | 1/Simpson | Richness | Coverage |
| **LH** | - | - | - | - | - | 119.50 | 1.83 | 2.76 | 99 | 83% | 36.75 | 1.77 | 4.48 | 33 | 90% |
| **BDH** | - | - | - | - | - | 100.00 | 3.50 | 15.75 | 100 | 99% | 44.75 | 1.30 | 2.07 | 44 | 98% |
| **HH** | - | - | - | - | - | 81.33 | 2.89 | 8.90 | 78 | 96% | 35.50 | 0.98 | 1.60 | 34 | 96% |
| **YR** | 47.00 | **2.37** | **7.26** | 42 | 89% | 76.00 | 2.77 | 9.62 | 69 | 91% | 58.33 | 2.96 | 13.17 | 58 | 99% |
| **SYH** | 18.00 | 0.95 | 2.15 | 18 | 100% | 108.33 | 1.65 | 2.65 | 63 | 86% | 41.20 | 1.42 | 2.93 | 37 | 90% |
| **BCYH** | 17.50 | 1.18 | 2.47 | 17 | 97% | 66.11 | 1.81 | 2.87 | 60 | 91% | 45.75 | 2.09 | 5.43 | 42 | 92% |
| **CJ** | 76.88 | **2.59** | **9.02** | 70 | 91% | 92.75 | 2.21 | 5.62 | 79 | 85% | 54.00 | 1.57 | 3.14 | 47 | 87% |
| **JJ** | - | - | - | - | - | 49.14 | 2.04 | 5.08 | 44 | 90% | 46.75 | 1.56 | 2.92 | 43 | 92% |
| **OJ** | 28.00 | 1.49 | 3.35 | 25 | 89% | 144.00 | 2.21 | 6.92 | 49 | 83% | 48.00 | 1.78 | 3.27 | 45 | 94% |
| **MJ** | 71.11 | **1.89** | **3.04** | 65 | 91% | 129.60 | 2.59 | 6.33 | 116 | 90% | 54.75 | 2.50 | 8.77 | 54 | 99% |
| **MLX** | 33.50 | 0.33 | 1.12 | 31 | 93% | 101.46 | 2.33 | 5.86 | 91 | 90% | 60.60 | 2.11 | 5.53 | 55 | 91% |
| **JLJ** | - | - | - | - | - | 95.00 | 2.24 | 4.21 | 89 | 94% | 48.00 | 2.27 | 6.49 | 47 | 98% |
| **YFX** | - | - | - | - | - | 77.60 | 2.84 | 11.43 | 71 | 91% | 61.50 | 1.53 | 2.25 | 59 | 96% |
| **ZJ** | 56.75 | 1.21 | 1.73 | 56 | 99% | 114.35 | 2.83 | 8.26 | 109 | 95% | 46.33 | 1.58 | 2.86 | 43 | 93% |
| **YJ** | - | - | - | - | - | 60.63 | 1.90 | 3.88 | 58 | 95% | 15.00 | 1.10 | 2.34 | 15 | 100% |
| **NLJ** | 24.00 | 0.58 | 1.26 | 24 | 100% | 82.00 | 2.10 | 5.14 | 70 | 85% | 16.00 | 1.69 | 3.11 | 16 | 100% |

**Table S4** Correlation analysis between the α-diversity indices of comammox and environmental factors. *P < 0.05; **P < 0.01

| **Parameter** | **Shannon** | **1/simpson** | **Chao1** | **Richness** |
| --- | --- | --- | --- | --- |
| **Shannon** | - | - | - | - |
| **1/Simpson** | **0.913**** | - | - | - |
| **Chao1** | **0.698*** | 0.584 | - | - |
| **Richness** | 0.648 | 0.534 | **0.993*** | - |
| **Salinity** | **-0.768*** | -0.588 | **-0.717*** | -0.663 |
| **pH** | 0.339 | 0.390 | -0.369 | -0.438 |
| **Temper** | -0.271 | -0.038 | -0.097 | -0.071 |
| **Particle size** | 0.083 | 0.168 | -0.340 | -0.333 |
| **Silt** | 0.128 | -0.207 | 0.544 | 0.558 |
| **Sand** | -0.056 | 0.229 | -0.426 | -0.438 |
| **Clay** | -0.032 | -0.221 | 0.234 | 0.232 |
| **WC** | 0.024 | -0.176 | 0.539 | 0.537 |
| **NH_4_^+^** | 0.224 | 0.107 | -0.060 | -0.027 |
| **NO_2_^-^** | -0.180 | -0.097 | -0.256 | -0.220 |
| **NO_3_^-^** | 0.222 | 0.360 | -0.105 | -0.075 |
| **Fe^2^** | 0.292 | 0.185 | 0.276 | 0.251 |
| **Fe^3^** | -0.276 | -0.449 | 0.389 | 0.409 |
| **TN** | 0.051 | -0.062 | 0.583 | 0.606 |
| **TC** | **0.763*** | **0.754*** | 0.438 | 0.449 |
| **TOC** | -0.160 | -0.271 | 0.472 | 0.501 |

**Table S5** Bioenv analysis of environmental factors and comammox community (OTU)

| bioenv |  |  |  |  |  |  |  |  |  | size | correlation |
| --- | --- | --- | --- | --- | --- | --- | --- | --- | --- | --- | --- |
| TOC |  |  |  |  |  |  |  |  |  | 1 | 0.5277 |
| NH_4_^+^ | TOC |  |  |  |  |  |  |  |  | 2 | 0.556 |
| Salinity | NH_4_^+^ | TOC |  |  |  |  |  |  |  | 3 | 0.6067 |
| Salinity | WC | NH_4_^+^ | TOC |  |  |  |  |  |  | 4 | 0.6044 |
| **Salinity** | **Particle size** | NH_4_^+^ | **Fe^3+^** | **TOC** |  |  |  |  |  | **5** | **0.6345** |
| Salinity | pH | Particle size | NH_4_^+^ | Fe^3+^ | TOC |  |  |  |  | 6 | 0.5861 |
| Salinity | pH | Particle size | NH_4_^+^ | Fe^2+^ | Fe^3+^ | TOC |  |  |  | 7 | 0.5689 |
| Salinity | pH | Particle size | WC | NH_4_^+^ | Fe^2+^ | Fe^3+^ | TOC |  |  | 8 | 0.5503 |
| Salinity | pH | Particle size | WC | NH_4_^+^ | Fe^2+^ | Fe^3+^ | TC | TOC |  | 9 | 0.5238 |
| Salinity | pH | Particle size | WC | NH_4_^+^ | NO_2_^-^ | Fe^3+^ | TN | TC | TOC | 10 | 0.4942 |

**
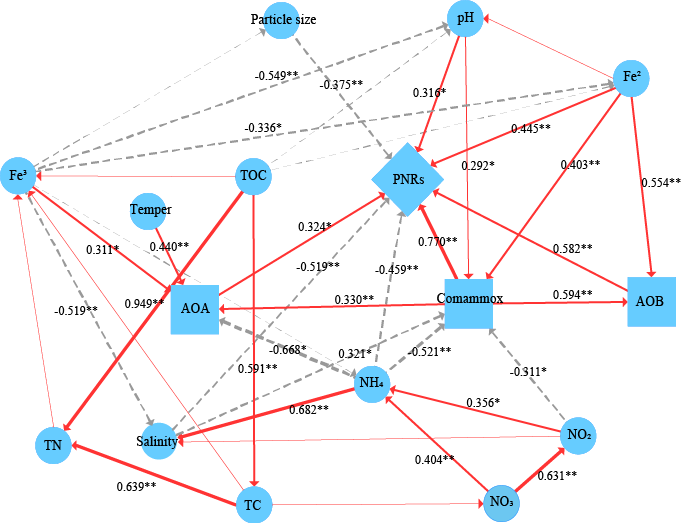
**

**Fig. S1** Pearson correlation analysis of environmental factors with ammonia-oxidizers abundance as well as PNRs.

(*P < 0.05; **P < 0.01）


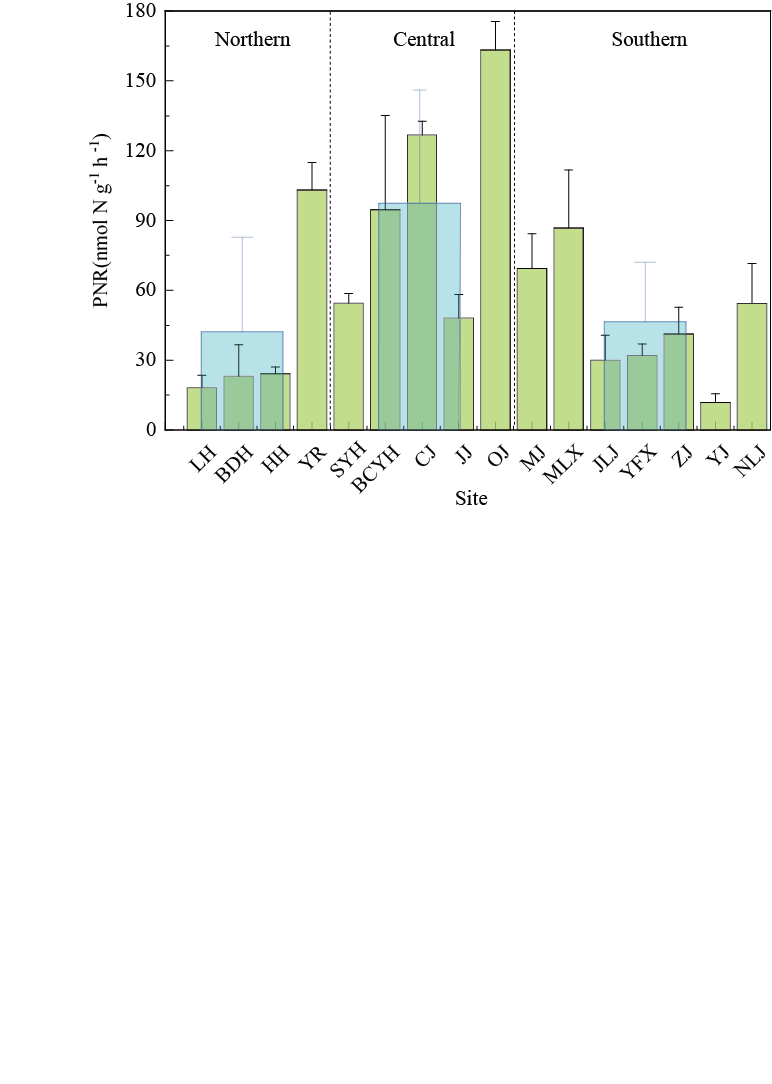


**Fig. S2** PNRs in the sediments of the estuarine tidal flat wetland. The error bars: standard deviations of triplicate incubations; Green bars: single samples; Blue bars: Average from different regions.

**
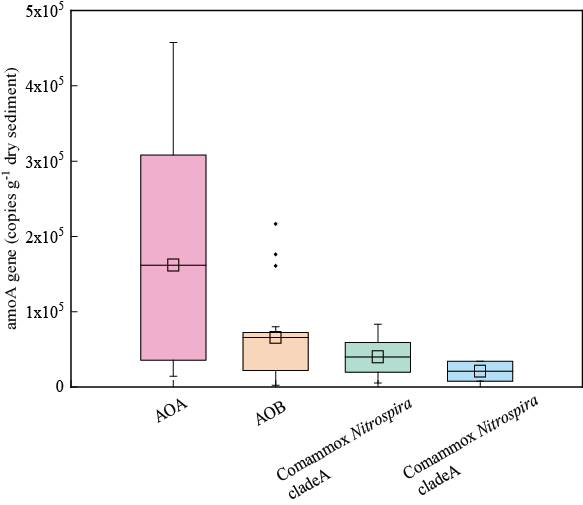
**

**Fig. S3** Abundance of ammonia-oxidizers in the coastal wetlands of China. The error bars: standard deviations of triplicate; line: average values; Splashes: outliers.

**
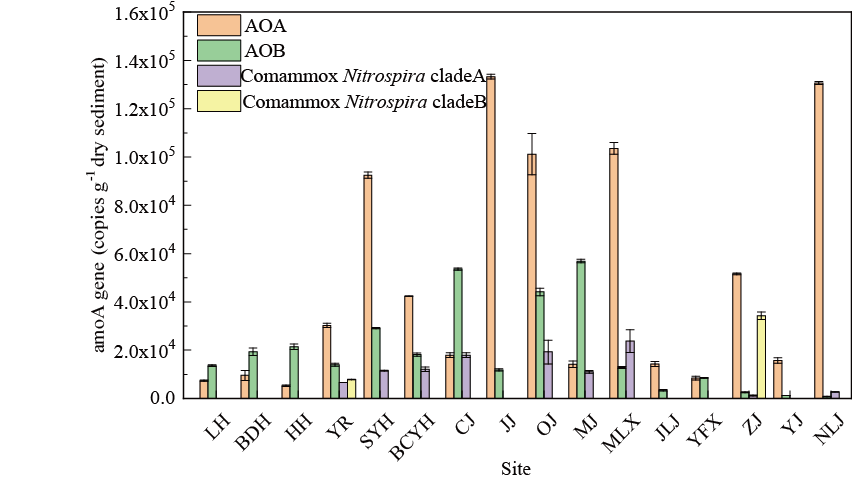
**

**Fig. S4** Gene abundances of ammonia-oxidizers based on the qPCR results. The error bars: standard deviations of triplicate.

**
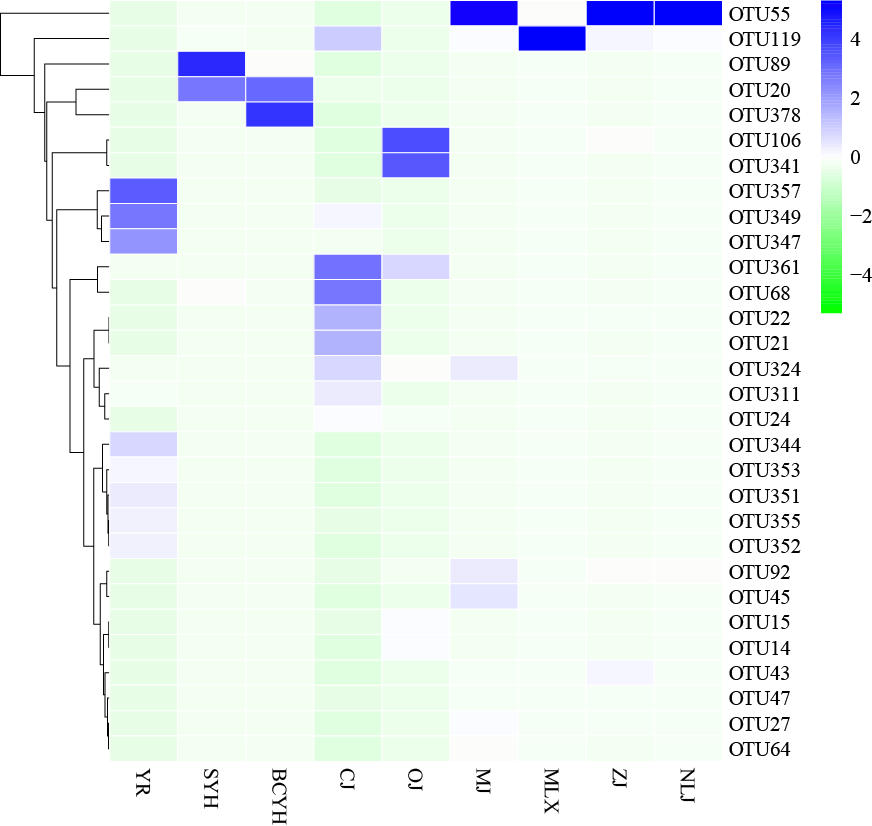
**

**Fig.S5** Heatmap of TOP 30 comammox OTUs

**
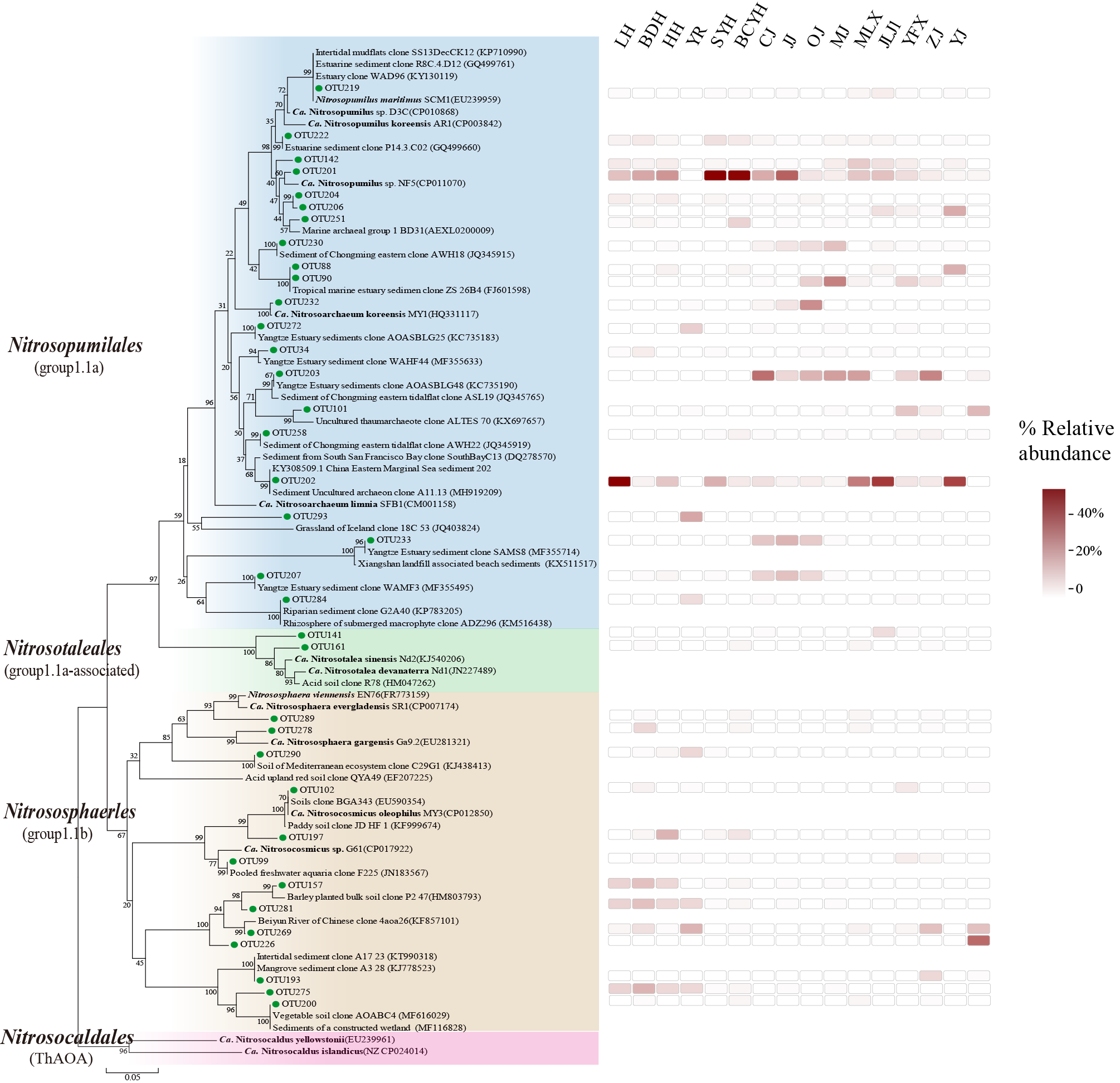
**

**Fig. S6** Phylogenetic tree of AOA *amoA* genes constructed by neighbor joining methods. Sequences were collected from this study and the GenBank database. Bootstrap values are given on the branches of the neighbor-joining tree. The scale bar represents 5% nucleic acid sequence divergence.**
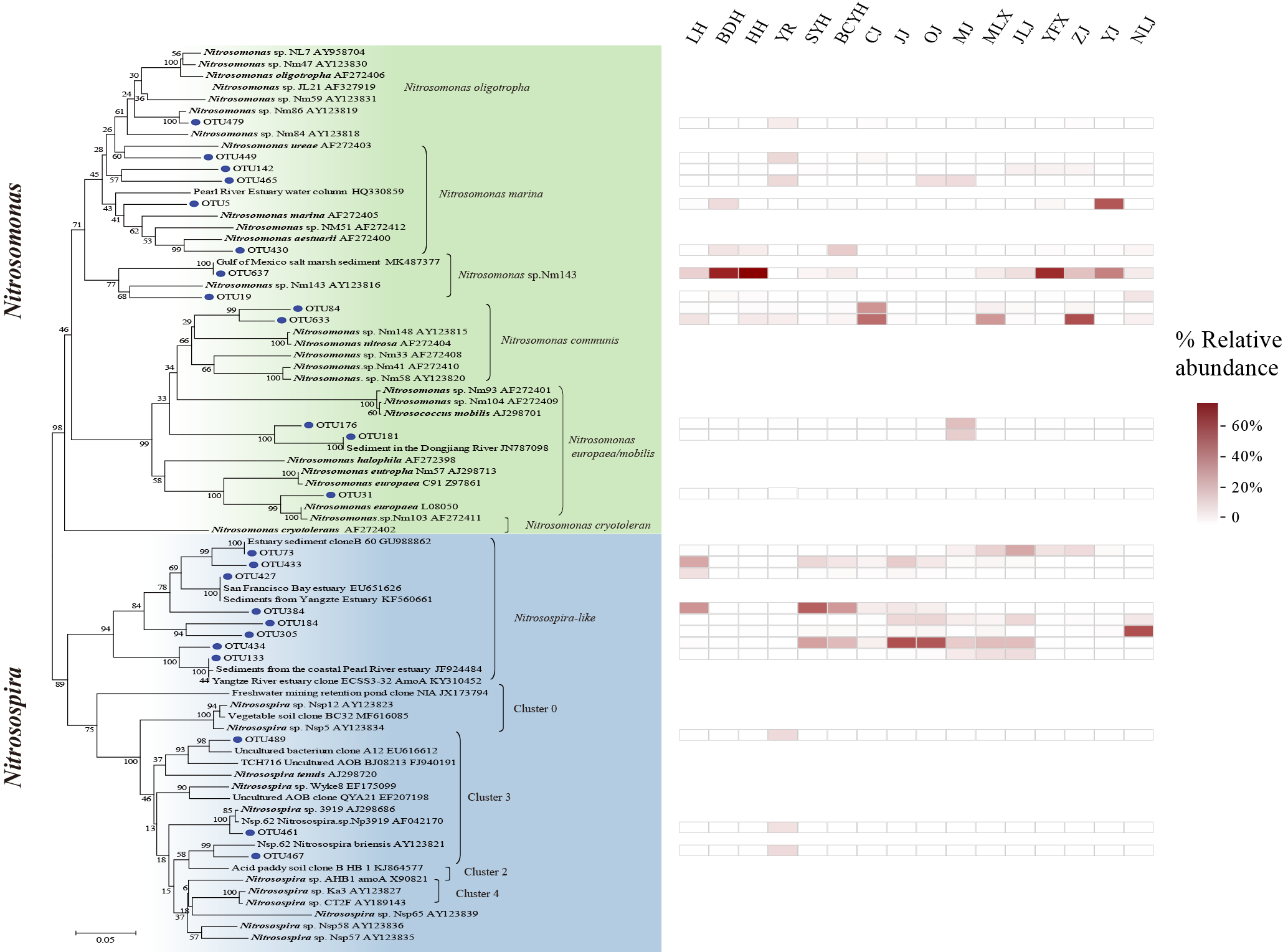
Fig. S7** Phylogenetic tree of AOB *amoA* genes constructed by neighbor-joining methods. Sequences were collected from this study and the GenBank database. Bootstrap values are given on the branches of the neighbor-joining tree. The scale bar represents 5% nucleic acid sequence divergence.

**References**

Yu, C., Hou, L., Zheng, Y., Liu, M., Yin, G., Gao, J., et al. (2018). Evidence for complete nitrification in enrichment culture of tidal sediments and diversity analysis of clade a comammox *Nitrospira* in natural environments. *Appl. Microbiol. Biot.* 102, 9363-9377.doi：10.1007/s00253-018-9274-0

Pjevac, P., Schauberger, C., Poghosyan, L., Herbold, C. W., Van Kessel, M. A., Daebeler, A., et al (2017). AmoA-Targeted Polymerase Chain Reaction Primers for the Specific Detection and Quantification of Comammox *Nitrospira* in the Environment. *Front.Microbiol*. 8, 1508. doi:10.3389/fmicb.2017.01508

Rotthauwe, J. H., Witzel, K. P., and Liesack, W. (1997). The ammonia monooxygenase structural gene amoA as a functional marker: molecular fine-scale analysis of natural ammonia-oxidizing populations. *Appl. Environ. Microbiol*. 63,470-4712. doi:10.1128/aem.63.12.4704-4712.1997

Pester, M., Rattei, T., Flechl, S., Grongroft, A., Richter, A., Overmann, J., et al. (2012). AmoA-based consensus phylogeny of ammonia-oxidizing archaea and deep sequencing of amoA genes from soils of four different geographic regions. Environ. Microbiol, 14, 525-539. doi:10.1111/j.1462-2920.2011. 02666.x
